# The Impact of Micro-Nanoparticles on Morphology, Thermal, Barrier, Mechanical, and Thermomechanical Properties of PLA/PCL Blends for Application in Personal Hygiene: A Review

**DOI:** 10.3390/polym17172396

**Published:** 2025-09-02

**Authors:** Tiisetso Ephraim Mokoena, Lesia Sydney Mokoena, Julia Puseletso Mofokeng

**Affiliations:** Department of Chemistry, University of the Free State (QwaQwa Campus), Kestell Road, QwaQwa, Phuthaditjhaba 9866, South Africa; mokoenatiisetsoephi@gmail.com (T.E.M.); mokoenals@ufs.ac.za (L.S.M.)

**Keywords:** biodegradable polymers, green biocomposites, PLA/PCL blends, phase separation, micro-nanocomposites, personal hygiene

## Abstract

This present review aims to provide a clear overview of the environmental impact of non-biodegradable materials, and the use of biodegradable materials as their replacements. Non-biodegradable polymers have been used in the past, and now they are considered a threat to the environment. Recently, it has become a necessity to manufacture products with biodegradable polymers to alleviate waste pollution because they can degrade in a short period of time in the environment. Biodegradable polymers can be used in various applications like cosmetics, coatings, wound dressings, gene delivery, and tissue engineering scaffolds. Blending biodegradable polymers could provide an excellent opportunity to produce innovative green biocomposites suitable for any desired applications. This paper reviews all the recent related works on biodegradable PLA and PCL materials and the introduction of fillers for the development of green biocomposites. The properties and characterisation of PLA/PCL blends and PLA-PCL-filler green biocomposites on morphology, thermal, mechanical, thermomechanical, and barrier properties are thoroughly reviewed. The applications, limitations, and future prospects of these green biocomposites is also highlighted.

## 1. General Introduction

Personal hygiene can be explained as the practice of maintaining the cleanliness of our body to prevent illness and the spread of diseases [1]. Products such as face masks, sanitary pads, wound dressings (bandage-like material), diapers for children and adults, and flushable and non-flushable wipes are known as personal hygiene materials. These products have proved reliable and given people comfort through the years, especially women, children, and sick people [2,3]. Most of them are manufactured from various synthetic polymers, such as polypropylene (PP), polyethylene terephthalate (PET), and/or the combination of high-density polyethylene (HDPE) and PP [4]. However, these polymer products (PP, PET, and HDPE) used in the health and hygiene field are a major source of waste pollution. The excessive use of personal hygiene products contributes to sanitary waste pollution because they are discarded into the environment after use. As a result, they remain intact in the ecosystem over a lengthy period due to their materials’ resilience, meaning that they are resistant to degradation [5,6,7]. The disposal of waste (personal hygiene products) via both illegal landfill dumping and uncontrolled incineration poses a serious environmental issue [8,9]. For instance, waste sanitary products disposed of via uncontrolled or illegal dumping are associated with environmental pollution such as landfill accumulation and underground water pollution [6,8]. Landfill accumulation is associated with continuous waste, land dumping, and underground water pollution (leaching of organic, inorganic, and other various toxic substances of concern found in waste plastic products) [8]. Heavy metals and other pollutants from waste damping sites are released via the ground and through the soil, affecting food crops and water, leading to health effects and issues for animals, plants, and human life [8,10], whereas uncontrolled incineration may lead to the formation of harmful chemicals, as it releases toxic emissions such as nitrous oxide, carbon dioxide, and sulphur dioxide, which are common greenhouse gases that contribute to global warming, as well as harming air quality [6,8,10].

Personal hygiene waste is also a major cause of solid sewer waste blockages because these materials are flushed away after use. As a result, they absorb food waste, human hair, cleaning agents, and blend with grease that people pour down the drain [1,2,11]. The blended sewer wastes then settle in sewer pipes, and over time they solidify, accumulate, and block the pipes. The blockage then causes the pipe to burst, which results in sewage flowing [2,11]. Municipality wastewater officials then spend public funds to remove and rectify blockages and repair damaged sewer pipes caused by flushed materials [11]. The flow of sewage poses serious health issues to humans due to exposure to sewer gas (hydrogen sulphide, ammonia, and methane). When this gas, which is known to be very toxic, is inhaled in sufficient quantities, it can lead to hydrogen sulphide poisoning. This hydrogen sulphide gas can cause dizziness, headache, cardiac irregularities, insomnia, respiratory distress, and irritation to the eyes [1,11,12]. It is imperative to establish proper waste management by maintaining and promoting cleanliness in the world.

Environmental waste pollution caused by petroleum-based polymers from single-use/disposable materials can be remediated using biodegradable polymers [13]. Biodegradable polymers are materials that are susceptible to biological degradation and can be used for a pre-determined time before degrading. These polymers are produced from renewable (plants and microbes) and petroleum resources (natural gas and coal). They offer end-of-life solutions through biodegradability or natural breakdown and composting, leading to a reduction in plastic pollution, and they are environmentally friendlier than petroleum-based polymers. Biodegradable polymers are essential because they are anticipated to reduce and overcome the impact of petroleum-based waste pollution on the environment [13,14,15,16]. Table 1 focuses on a comparison between biodegradable and non-biodegradable polymers.

**Figure 1:**
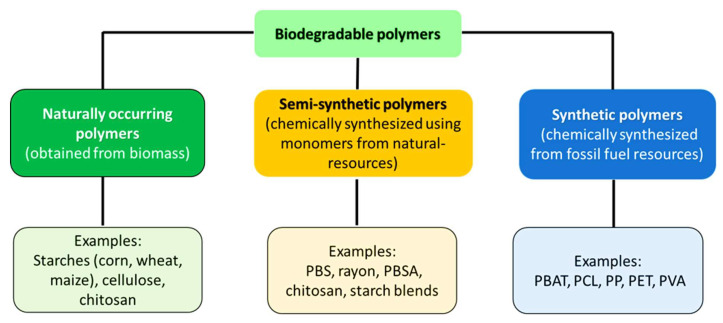
Classification of biodegradable polymers based on their origin in detail.

Biodegradable polymers can be classified into three categories, namely, natural, semi-synthetic, and synthetic [15,18,19]. Natural biodegradable polymers are produced from biomass (plants, animals, and microorganisms) such as polysaccharides (starch, cellulose, lignin). Semi-synthetic polymers are derived from natural products, but they are chemically modified to improve their properties. For instance, polymers like polylactic acid (PLA), polybutylene succinate (PBS), and starch blends are chemically synthesised from natural resources. Furthermore, cellulose in its natural state can be chemically treated to form cellulose acetate (CA) and microcrystalline cellulose (MCC). Synthetic biodegradable polymers are known as man-made polymers and are produced through chemical synthesis from petrochemical resources, with examples including poly(butylene succinate terephthalate) (PBAT), polyethylene terephthalate (PET), polypropylene (PP), polyvinyl alcohol (PVA), and polycaprolactone (PCL) [15,17,18,19,20,21]. These classifications and their examples are outlined in Figure 1.

Biodegradable polymers have been extensively researched by both industrial and academic researchers due to their exceptional properties, namely, low toxicity, environmental friendliness, chemical adaptability, biodegradability, and biocompatibility [5,16,22]. These materials can degrade into water (H_2_O), carbon dioxide (CO_2_), and biomass, which are non-toxic products, and they can decompose when exposed to naturally occurring bacteria [14,15,16]. A number of studies have been conducted on biodegradable polyesters. Polyesters are materials that have ester groups in their repeat units that are susceptible to hydrolytic cleavage, and they play a key role in biodegradable plastics. They are made up of two main groups of aliphatic and aromatic polyesters [21,22,23,24,25,26]. There are several biodegradable polyesters that have been produced to substitute petroleum-based polymers. These polymers include poly (lactic acid) (PLA), poly (ε-caprolactone) (PCL), polyvinyl alcohol (PVOH), polypropylene carbonate (PPC), polyhydroxy alkanoates (PHAs), including polyhydroxybutyrate (PHB), polyhydroxyvalerate (PHV), and co-polymer poly (3-hydroxybutyrate-co-3-hydroxyvalerate) (PHBV) [21,24]. Among the above-mentioned biodegradable polymers, PLA and PCL have demonstrated potential to mitigate waste pollution of personal hygiene products [24,25,27]. These two polymers have been utilised and explored for their wide range of applications, especially in packaging (compostable bags), agricultural (mulch films), textile (sanitary napkins), and biomedical (degradable sutures) applications. Their combination creates a novel material that can go beyond their unique individual capabilities, and they can be blended effectively to exhibit exceptional properties. Further, the properties of PCL (high thermal stability, good processability, and high elongation at break) can be added to PLA to improve its fluidity, flexibility, and impact resistance, and that can broaden their uses in manufacturing and research purposes [25,26,27].

### 1.1. Poly (Lactic Acid) (PLA) Background

**Figure 2:**
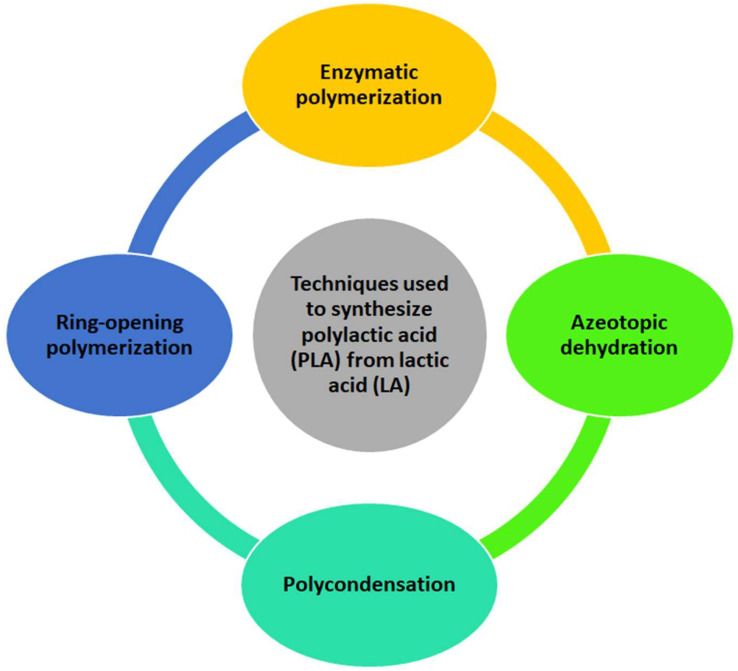
Techniques used to synthesise polylactic acid (PLA) from lactic acid (LA).

PLA is an aliphatic polyester derived from the lactic acid (LA) monomer, and it consists of repeating lactic acid units linked through ester bonds. This polymer is produced via chemical synthesis or by fermentation of sugars like glucose, maltose, and dextrose from corn and potato starch. The degradation mechanisms of PLA entail various mechanisms that contribute to its breakdown. Hydrolysis, enzymatic, thermal, and microbial mechanisms are examples of PLA degradation mechanisms. For instance, PLA undergoes hydrolysis degradation through the cleavage of ester groups by water [13,28,29,30,31]. It is produced using a multi-step process that begins with lactic acid (LA) and polymerisation. It can be synthesised from LA using ring-opening, enzymatic, azeotropic, and polycondensation polymerisation (Figure 2) [23,30,32].

**Figure 3:**
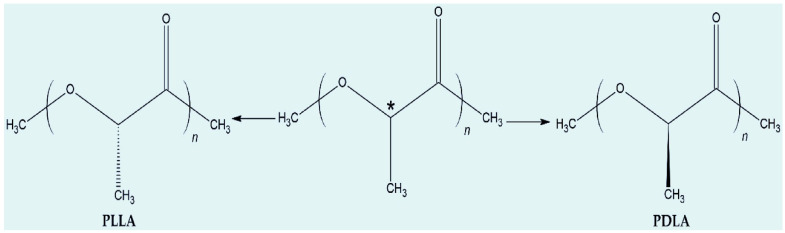
Illustration of PLA showing two stereochemical forms, poly (l-lactide) (PLLA) and poly (d-lactide) (PDLA) [28]. (Open access from ref. [28]. Copyright 2023, Elsevier).

PLA is a rigid polymer, and it is brittle below its glass transition temperature (T_g_), ranging from 50 to 70 °C, with a melting temperature ranging between 150 and 180 °C [13,33,34,35]. It exists in two main stereochemical forms, poly (l-lactide) (PLLA) and poly (d-lactide) (PDLA), due to the asymmetric carbon atom in its molecule, and these forms are mirror images of each other. Both PLLA and PDLA can be synthesised via direct polycondensation and ring-opening polymerisation of lactic acid [13,23,30]. The isomeric forms of PLA (L- and D-) are illustrated in Figure 3, and they determine the properties of the polymers; note that both L-lactide and D-lactide produce a semi-crystalline polymer [22,28,29]. In addition, racemic D, L-lactide is another stereochemical form, having equal amounts of D- and L-lactic units (50:50 mixture). Each lactic acid unit contains one chiral centre at each lactic acid at the α-carbon, whereby L-lactide has an (S)-configuration and D-lactide has an (R)-configuration. The stereochemistry of D, L-lactide is important as it directly affects or controls polymer properties (crystallinity, degradation rate, and melting behaviour) and optimisation of applications in various fields. For instance, racemic D, L-lactide (PDLLA) is an amorphous polymer with low crystallinity that has good transparency and flexibility. It is suitable for applications demanding flexibility and faster degradation rates (e.g., packaging). This racemic mixture is produced when chemical synthesis is used, while enantiomerically pure lactic acid (L-lactide or D-lactide) is produced by microbial fermentation [13,22,30,36,37].

PLA has high stiffness and tensile strength, and good rigidity. Its elongation at break is lower than 10% and it has a poor impact strength that is close to 2.5 kJm^−2^ [26,29,33,38,39]. The advantage of PLA is that it is very versatile and can be moulded into several structures depending on the application desired. For instance, it can be moulded into flexible films, bottles, and rigid packaging [28,38]. It can also be used in biodegradable fabric items like wipes, diapers, and feminine hygiene products [22,38,40]. Despite its advantages, it has some significant shortcomings, including high brittleness; poor thermal stability, toughness, and barrier permeability properties; and a slow crystallisation rate during production, which hinders its industrial use. Moreover, there are processing challenges related to PLA, especially above its melting temperature (180 °C), meaning that when exposed to elevated temperatures, it breaks down [22,41,42,43]. These problems have made researchers come up with methods that could improve the poor properties of PLA. In this regard, various methods, like polymer blending, copolymerisation, plasticiser use, and reinforcing fillers, have been employed to solve and minimise the shortcomings of PLA [18,26,42,43,44].

### 1.2. Poly (ε-Caprolactone) (PCL) Background

**Figure 4:**
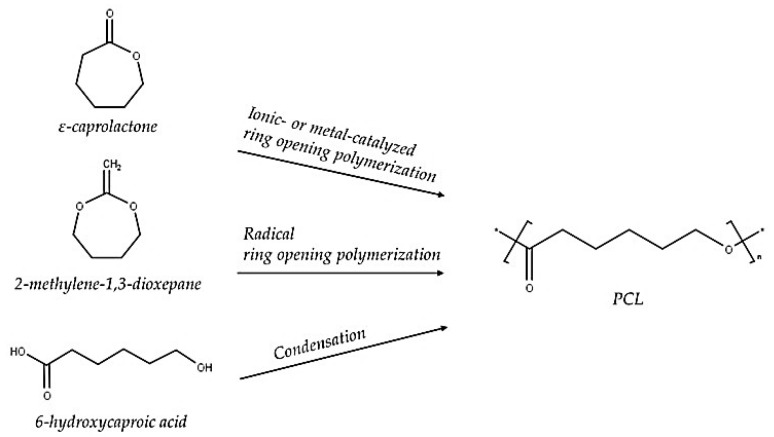
Illustrates the synthesis of PCL by radical ring-opening polymerisation [46]. (Open access from ref. [46]; copyright 2022, MDPI).

PCL is a semi-crystalline aliphatic polyester that has high flexibility and toughness. It is produced by radical ring-opening polymerisation and ring-opening by cationic, anionic, or metal catalysts from a caprolactone monomer (Figure 4). Its chains undergo disintegration and biodegradation through hydrolytic or enzymatic reactions. The thermal stability of PCL begins around 390–420 °C, and its degradation produces the caprolactone monomer, carbon dioxide, and water. The degradation mechanism of PCL occurs through the cleavage of the ester groups, and at higher temperatures it can undergo depolymerisation, which can yield its caprolactone monomer. PCL has a low glass transition temperature (T_g_) of around −60 °C, and a low melting temperature ranging between 55 and 70 °C [45,46,47,48]. PCL has several advantages including high flexibility that gives it a high elongation at break; biocompatibility; good ductility, caused by its low glass transition temperature; good moulding ability; and easy processability due to its low melting temperature. It is an ideal candidate material for use in the biomedical field as a controlled drug delivery system, in long-term implants, and in packaging because of its flexibility, high water resistance, and biocompatibility. Despite its excellent properties, PCL has some disadvantages, including a low melting temperature and slow degradation rate, and it is still reasonably costly to synthesise or purchase [17,21,35,38,39]. Although PCL has attractive properties, its lower melting temperature prohibits its use as an individual polymer. Therefore, blending PCL with other higher-melting-point polymers might extend its use in different applications requiring its properties and a higher melting temperature.

### 1.3. Polymer Blending

Polymer blending is considered the most convenient and simple way to utilise the properties of individual polymers. This method is a novel approach because it can improve the properties of individual polymers by combining their merits to develop new and improved materials with customised properties to fit the specifications of the desired application [24,49,50]. Blending offers advantages such as cost-effectiveness and is less time-consuming than developing new polymers. The main objective of preparing a blend of two or more polymers is to improve and maximise the material performance by capitalising on the good original properties of each component in the blend [50,51]. In other words, the properties of the blends can be manipulated by selecting a suitable polymer with specific attributes (viscosity, polarity, biocompatibility, degree of crystallinity, and molecular weight). For example, PLA is a brittle polymer with a lower thermal degradation temperature, and its blend with PCL reduces its brittleness and improves its thermal stability and mechanical properties, while PCL’s melting temperature and other properties could also be improved to enable the usage of these polymers in a variety of applications [52,53,54,55]. During blending, there are several very important factors that must be considered, such as blend composition, mixing temperature, time, and rotational speed. These factors should be considered because they affect how polymers interact with each other during processing [24,51]. Blending PLA with PCL has proven to be the most effective way to improve resistance to stress cracking and toughness, and impacts the strength of PLA [55,56,57]. Whilst PCL is not a bio-based polymer, it is known for its biodegradability and biocompatibility. These traits make it a suitable polymer to be blended with PLA because both the biodegradability and biocompatibility of these polymers will be preserved [58]. However, the main challenge faced by polymer blends in general is the incompatibility between two or more polymers. It is known from the literature that most polymers are thermodynamically immiscible, which leads to phase-separated morphologies over a wide range of compositions [41,56,59,60,61,62]. This factor could be characterised by high interfacial tension and poor adhesion between polymers, thereby affecting the overall performance of the blends [62]. From this perspective, the full benefits of blending these two polymers cannot be realised due to the immiscibility between PLA and PCL, which is caused by their different properties, such as surface energies and viscosities [62,63]. Thus, incorporating a third component in this system, such as a compatibiliser, reinforcing filler, or chemical modifier, could reduce surface interfacial tension between the PLA/PCL phase [58,64,65,66]. The focus of the present work is on reviewing the addition of fillers to polymer blends, because they are able to reduce interfacial tension while improving the adhesion between the two phases, as mentioned above. The performance of the polymer blend composites relies on the efficiency of fillers when they interact with the polymer blends. For that reason, fillers could selectively localise in the interface between the two phases, locate in the individual polymer phase, or localise in both polymer phases simultaneously [50,60,67,68].

Presently, there is a tremendous interest in using nanomaterial fillers to stabilise the interface between immiscible polymers in blends to improve their properties [34,53,60,66,67,69,70]. According to the literature, nanomaterials like silicon dioxide (SiO_2_), montmorillonite (MMT), calcium carbonate (CaCO_3_), zinc oxide (ZnO), silver nanoparticles (AgNPs), and titanium dioxide (TiO_2_) can enhance the properties of the polymer blends they are incorporated into. Those properties include the surface morphology of the polymer blends; antibacterial, thermal stability, and barrier properties; and mechanical and thermomechanical properties [34,70,71,72,73,74]. The versatility of these nanomaterials has been shown to broaden the application of polymer blends in various fields like tissue engineering, drug delivery, cosmetics, packaging films, rubber products, and in paints [53,70,72,73]. However, the application of interest in this review is personal hygiene materials. Based on the properties of the above-mentioned fillers, they have the ability to address the issue of environmental waste pollution caused by personal hygiene products. The properties and different types of fillers and their applications are also introduced in the following section.

## 2. Filler Types, Properties, and Their Versatile Uses

Fillers made from inorganic materials possess excellent features like non-toxicity, thermal stability, mechanical reinforcements, and antibacterial properties. They have the potential to be used in personal hygiene applications due to their ability to combat bacterial threats. It is well known that polymers lack antibacterial capabilities, which limits their usage in hygiene applications [70,72,75,76]. Common inorganic fillers include silica (SiO_2_), calcium carbonate (CaCO_3_), zinc oxide (ZnO), silver nanoparticles (AgNPs), and titanium dioxide (TiO_2_) [53,64,70,71,74,75]. Organic compounds are another nanomaterial of interest; they are carbon-based materials that have a large surface area, high electrical and thermal conductivity, and good mechanical properties, like carbon nanotubes, graphene, and carbon black. These materials can be used in various fields like energy storage, electronics, aerospace engineering, automobiles, and biomedicine [77]. However, organic materials are more volatile and flammable in comparison to inorganic materials, and their suitability rests on the application of choice. Cellulose is the most available organic natural biopolymer, and it is obtained from natural sources such as wood and plant fibres. It can be converted to microcrystalline, bacterial, and nanocrystalline cellulose. Microcrystalline cellulose (MCC), as a derivative of cellulose, can provide high thermal stability, mechanical reinforcement, and barrier properties to polymer composites. It is biodegradable, nontoxic, and biocompatible, which makes it useful for the development of green biodegradable polymer composites [3,78,79]. This is a versatile material that can be applied in various fields, especially in personal hygiene care products (diapers, feminine care items) and in biomedical materials (wound dressings and face masks) [3]. The combination of inorganic and organic fillers could be used to fabricate exceptional polymer blend micro-nanocomposites with new balanced properties that would increase their performance in a variety of fields [80]. The properties and applications of different fillers are tabulated in Table 2.

The correlations between morphology, thermal, thermomechanical, mechanical, and barrier properties will be examined for polymers, polymer blends, and polymer blend micro-nanocomposites. Applications and future prospects will also be touched on.

## 3. Morphology

Morphology is defined as the physical structure and arrangement (size and shape) of polymers. Knowledge of morphology is very important because it describes how polymers interact and behave. Polymers have regions that are either crystalline or amorphous; these can be blended to enhance their properties. Polymer blends are generally composed of two or more components, and these blends can either be homogeneous (miscible) or heterogeneous (immiscible) [88,89,90,91]. Notably, some factors influence the morphology of polymer blends and/or blend micro-nanocomposites, so understanding them is essential because they often dictate the functional properties of blends [92]. The section below summarises those factors and explores their effect on the morphology of the polymer blends and/or blend micro-nanocomposites.

### 3.1. Factors That Influence the Morphology of Polymers, Their Blends, and Polymer Blend Micro-/Nanocomposites

**Figure 5:**
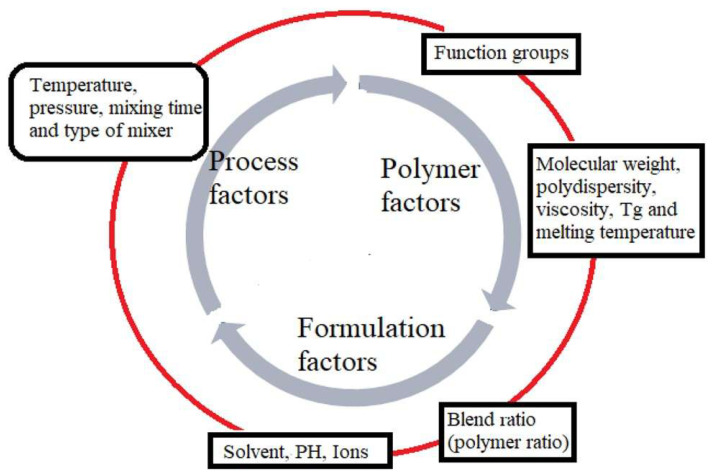
Illustration of the factors affecting the morphology of polymer–polymer interactions [92]. (Open access from ref. [92]. Copyright 2023, Springer Nature).

The morphology of polymers, their blends, and blend micro-/nanocomposites are affected or influenced by various factors such as the processing conditions applied (mixing time, rotation speed, and temperature), polymer factors (viscosity, melting temperatures, molecular weight, polarity), and formulation factors (blend ratios) (see Figure 5) [92,93,94].

Miscibility and compatibility in polymer blends are the main controlling factors that could significantly affect the properties and contribute to the performance of the polymer blend and/or blend micro-nanocomposites [49,95,96,97]. However, these two properties have different meanings. The miscibility of polymers can be explained as the behaviour of two polymers upon blending, forming a one-phase system or two phases, depending on the individual properties of the polymers and the compositions used. For example, for a miscible blend (one phase), a single glass transition temperature (T_g_) should be detected [98]. On the other hand, when two or multiple separate glass transition temperatures are obtained, the blend is immiscible (two separate phases). Further reasons for miscible and immiscible blends could be related to the thermodynamic miscibility conditions, and whether they are met upon mixing or blending. Meaning that if the Gibbs free energy (∆G_mix_) was negative (∆G_mix_ < 0), the blend would be miscible, and if the Gibbs free energy (∆G_mix_ > 0) was positive, the blend would be immiscible [95]. Compatibility is explained by how well the components in the polymer blends can interact with each other, and that defines the polymer blend properties. For instance, if phase separation is observed, the blend is incompatible, which can be seen by gaps in the interface caused by pull-outs and cracks. However, a blend can be phase separated and compatible, which could be caused by a lack of gaps in the interface, meaning no cracks and pullouts in the blend. The overall performance of polymer blends depends on the compatibility between the two components in the blend [49,95,97,98]. Surface energy determination is one of the methods used to indicate how compatible components are in the blend and/or blend composites. Incompatibility of the polymers is noticeable if the difference between the polar components of the materials’ surface free energies is too large. Whereas compatibility of the polymers is apparent when the materials’ surface free energies are small or the same. For that reason, the difference in surface energy of the polymers in blends could determine their compatibility, which will govern the performance of the blends [41,99]. Other factors that are involved in deciding the morphology and localisation of fillers in polymer blends and composites include degree of crystallinity, viscosity of polymers, and molecular weight, as mentioned above. In this section, the morphology of the polymers and their blends and/or blend micro-/nanocomposites is reviewed by exploring several techniques such as scanning electron microscopy (SEM), transmission electron microscopy (TEM), surface energy evaluation system (SEES), melt flow index (MFI), SEM–energy-dispersive spectroscopy (SEM-EDS) and atomic force microscopy (AFM) [41,88,100,101,102,103,104]. The morphologies of PLA/PCL blends and their blend micro-nanocomposites will be addressed in detail in the following section because this review targets the properties of PLA/PCL blend micro-nanocomposites, with the purpose of using this system in personal hygiene applications.

#### 3.1.1. Evaluation of Morphology of PLA, PCL, and Their Blends

**Figure 6:**
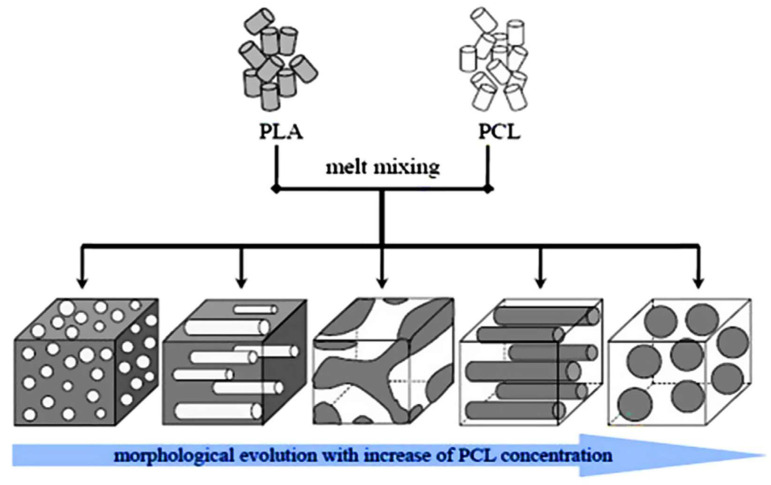
Morphologies of PLA/PCL blends showing different phases, droplets in the matrix, fibrous, co-continuous structure, lamellar, and/or ordered microphases [107]. (Reproduced with permission from ref. [107]. Copyright 2008 Elsevier).

Knowledge of polymer morphology is crucial because it provides a clear understanding of how polymers interact with each other and how one phase is distributed within the polymer matrix or vice versa [88,89]. Studies on the morphology of PLA have shown a smooth and compact surface, indicating its low toughness, high brittleness, and the absence of plastic deformation [27,102,105]. On the other hand, neat PCL shows a rough fractured surface, indicating its high toughness and no traces of the material being brittle [27,102]. Their blends can either be miscible or immiscible, and they may be compatible or incompatible depending on thermodynamic and kinetic factors. Most studies indicate that immiscibility between PLA and PCL is dominant irrespective of their relative composition contents [33,60,97,106]. The morphology of the PLA/PCL blends can consist of another polymer forming droplets in the matrix, fibrous morphology, co-continuous, lamellar, and/or ordered microphase morphology. As illustrated in Figure 6, each polymer structure possesses its own characteristics in relation to mechanical, thermal, and barrier properties [104,107,108]. Figure 6 illustrates how PLA/PCL blends behave when the PCL content increases in the blend.

**Figure 7:**
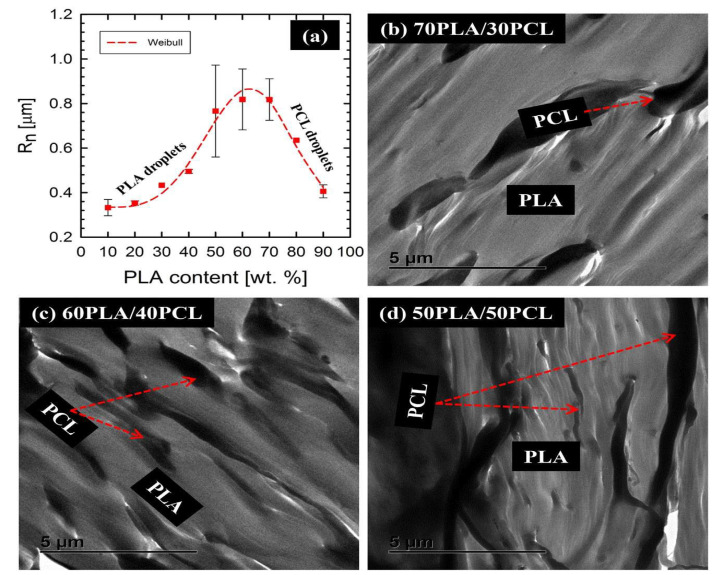
Illustrates (**a**) number-average sizes of the PLA and PCL droplets, and TEM micrographs of the PLA/PCL blends with different blend compositions of (**b**) 70/30, (**c**) 60/40, and (**d**) 50/50 wt.% [41]. (Reproduced with permission from ref. [41]. Copyright 2018 Elsevier).

As schematically illustrated in Figure 6 above, increasing the PCL content in the PLA matrix increases PCL’s particle size, resulting in changed/different phase structures. In this regard, Yeh and co-workers [109] evaluated the influence of miscibility and morphology of poly (lactic acid) (PLA)/poly(ε-caprolactone) (PCL) blends. A series of PLA/PCL blends (90/10, 85/15, 80/20, 70/30, 60/40, 50/50, 40/60, 30/70, 20/80 wt.%) were prepared by the melt-blending method, and the morphology of the blends was analysed by SEM. The Authors observed that the addition of PCL imparted its ductile property into the PLA matrix, making it less brittle. They further observed that when the PCL content increased above 30 wt.%, many traces of PCL pullouts were clearly seen. Furthermore, the dropout traces found between PLA and PCL at the fractured surface were caused by poor interfacial adhesion between the two polymers. The authors concluded that the combined results of the SEM morphologies, differential scanning calorimetry (DSC), and dynamic mechanical analysis (DMA) showed a partially miscible blend formed between PLA and PCL. This was based on the melting temperatures of PLA and PCL that shifted towards each other and the reduction of the glass transition temperatures (T_g_) observed by DSC and DMA, respectively. A different behaviour to Yeh et al. [109] was reported by Finotti and co-workers [106], who investigated immiscible poly(lactic acid)/poly(ε-caprolactone) blends with 95/5, 90/10, and 80/20 *w*/*w* compositions. The morphologies of fractured surfaces of PLA/PCL blends was examined by SEM. The authors observed a phase-separated morphology in all the proportions of PLA/PCL blends, indicating that PLA and PCL are immiscible with each other. In addition, increasing the PCL content increased the average particle size of PCL from 0.31 to 0.71 µm, respectively, for 5 and 20 wt.%. Research by Wachirahuttapong et al. [33] and Przybysz-Romatowska et al. [110] supports the findings of Finotti et al. [106] that PLA/PCL blends are immiscible, which suggests phase separation between PLA and PCL. This phenomenon can be influenced by thermodynamic parameters (entropy of mixing (∆S_m_), enthalpy of mixing (∆H_m_), and Gibbs free energy of mixing (∆G_m_)) and kinetic factors (viscosity of polymers, temperature, shear rate, and mixing time). From a thermodynamic standpoint, PLA/PCL blends are immiscible because the Gibbs free energy of mixing (∆G_mix_) upon blending is positive, signifying phase separation occurrence, resulting in an immiscible blend [60,95,110]. From a kinetic factor perspective, it determines how polymers behave during processing, thus affecting their final product properties. For instance, PLA/PCL blends are immiscible because of their high differences in viscosity ratios. In addition, the elasticity property of PCL could also affect the morphology of the PLA/PCL blends. PCL has a higher viscosity than PLA polymer; thus, the lower viscosity of PLA would result in PCL having large droplet sizes in the blends, which could cause coarse morphology that could lead to poor mechanical properties [41,60,111]. Botlhoko et al. [41] investigated melt-processed polylactide/poly(ε-caprolactone) blends. The authors provided new insights into the morphology of PLA/PCL blends with different compositions ((a) 90/10, (b) 80/20, (c) 70/30, (d) 60/40, (e) 50/50, (f) 40/60, (g) 30/70, (h) 20/80, and (i) 10/90 *w*/*w*), whereby SEM was used to examine the fractured surface of the materials. Phase-separated morphology was visible in all the different PLA/PCL blends, which indicated poor interfacial adhesion between the two polymers. The authors further explained that blends with a higher PLA content showed a higher average particle size droplet compared to the blends with a higher PCL content. The droplet sizes of PCL for 10, 20, 30, and 40 *w*/*w* were larger than those of PLA with the same compositions (see Figure 7a). This phenomenon was explained by differences in the viscosity ratio: as the viscosity of PCL is greater than that of PLA, the dispersed phase, in this case PCL, will have bigger droplets in size than PLA. For better visibility of the polymers in the blends (70/30, 60/40, and 50/50 *w*/*w* PLA/PCL), TEM was utilised. Phase separation morphology was detected in all of the blends, and this observation correlates with the SEM results. It was noted that for the 50/50 PLA/PCL blend, the change in morphology is observable from droplet morphology (where PCL has a lower content) to co-continuity morphology because of the higher PCL content in the blend. When a co-continuity morphology is observed, the two components form an interconnected phase. Figure 7b–d represent a co-continuous morphology, with the white colour showing the PLA region and the black colour showing the PCL with irregular size. It was concluded that the overall morphologies of all the blends showed that PLA and PCL are immiscible with each other. Notably, Table 3 summarises the information on the morphology of PLA/PCL blends that could not be included in the above discussions; however, it only draws attention to key points that complement the discussed research.

#### 3.1.2. Morphologies of PLA/PCL Blend Micro-Nanocomposites: Effect and Prediction of Filler Localisation on Polymer Blends During Processing

The concept of adding micro-/nano-sized fillers as a third component into immiscible and/or partially miscible polymer blends is beneficial and significant, because they can tailor the phase morphology of the polymer blends [101,114]. In a two-phase separated blend, this can be achieved when a filler is selectively localised in the main phase or localised in the minor phase and/or at the interface between the two phases [101,114,115]. Filler localisation is a significant property because it governs the morphology of the polymer blend micro-/nanocomposite by estimating the interactions between the two polymers and the filler [116]. For instance, if the filler is selectively localised in one of the polymeric components, it means that the affinity between the filler and the polymer component is very strong, which might lead to good dispersion of the filler in that polymer phase. However, it does not necessarily mean that when a filler has a strong affinity with the polymer component, the filler will disperse into that polymer. Sometimes, the filler particles might have a stronger affinity towards each other instead rather than the polymer, thus forming agglomerates in the polymer component. This behaviour can be further explained by the surface energies and polar characters of the fillers and the polymers. Suppose a filler’s surface energies and polar character are higher than those of both polymers in the blend. In that case, the filler will then migrate to the interphase between the two components, or it will form agglomerates [24,101,115,117]. Another method to predict filler localisation is by theoretical calculation of the wetting coefficient (ω_α_). This theory states that if ω_α_ > 1, the filler will preferentially locate within the polymer matrix, whereas if −1 < ω_α_ < 1, the filler will localise in the interface between the two phases, and lastly if ω_α_ < −1, the filler will preferentially localise in the dispersed phase [68,75,116]. In this context, Motloung and co-workers [60] investigated the morphologies of PLA/PCL blends and the prediction of cellulose nanocrystalline (CN) localisation in the blends. The authors evaluated the morphology of the PLA/PCL blend (70/30) and blend nanocomposites with 1, 2, 3, and 5 wt.% CN content by SEM analysis. A phase-separated morphology was observed with the blend, whereby the PCL phase formed droplets and dispersed in the continuous PLA phase, which indicates immiscibility between PLA and PCL, as described by other authors [33,41,106,110]. It was further observed that the droplet sizes of PCL in the blend were reduced upon incorporation of CN nanoparticles at lower contents (1 and 2 wt.%), which was determined by the image processing software ImageJ (NIH). This is an indication of improved compatibility between the blend phases, which resulted in better performance of the nanocomposites. However, increasing the CN nanoparticle content further resulted in similar behaviour to the neat blend, meaning that higher CN contents had no influence on the blend. The authors further explained the morphology by predicting filler localisation, which was achieved by determining the surface energies of the polymers and the filler. They also calculated the interfacial tensions of the blend (polymer–polymer, polymer–filler), which predicted the filler localisation in the blend (see Table 4). The wetting coefficient (ω_α_) is not shown in Table 4, but it was revealed in their discussion. PLA in this study was presented as polymer A, and PCL as polymer B, and the wetting coefficient (ω_AB_) was determined to be 1.15, which is greater than 1. This means that CN nanoparticles should preferentially localise in the PLA phase, which is seen by the calculated interfacial tension value of PLA/CN (1.97) that is close to that of PLA/PCL (2.20) in comparison to the PCL/CN value (4.5), which is very far from the blend. It is noteworthy that thermodynamic factors are not the only factors that influence filler localisation; kinetic factors also play a huge role in influencing filler localisation during processing. From this perspective, the reduction in PCL droplet size could be assumed to be due to CN nanoparticles being localised in the PCL phase during processing. For example, during processing, PCL melts first, and that could cover up the nanoparticles before they localise into the PLA phase, as predicted by the thermodynamic factors in Table 4. From this behaviour, the authors concluded that the filler localisation was controlled by kinetic factors instead of thermodynamic factors.

**Figure 8:**
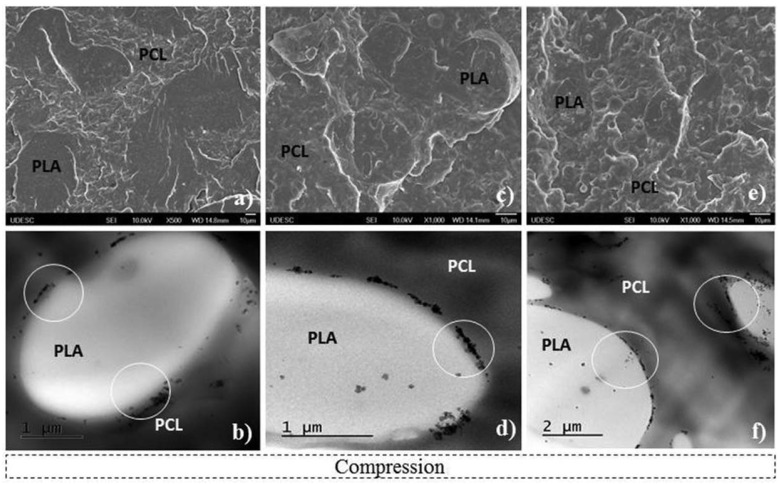
SEM images (**a**,**c**,**e**) and TEM images (**b**,**d**,**f**) of 42/58 *w*/*w* PLA/PCL blend [118]. For (**a**,**b**) 1.0; (**c**,**d**) 2.0; and (**e**,**f**) 3.0 wt.% of TiO_2_ content used, the circles indicate the interface between PLA and PCL, and the localization of titania. (Reproduced with permission from ref. [118]. Copyright 2017, John Wiley and Sons).

A study by Decol et al. [118] made a different observation with regards to the morphology with respect to the Motloung et al. [60] study. The authors investigated the interfacial localisation of titanium dioxide (TiO_2_) nanoparticles in PLA/PCL blends with SEM and TEM for better visualisation of nanoparticles. The remarks that were made about morphologies of the blends were that TiO_2_ nanoparticles were seen to be preferably localised at the interface between the PLA and PCL phases (Figure 8b,d,f). Furthermore, it was also noted that some of the nanoparticles were present in the PLA phase, as seen in Figure 8d, and that was due to a thermodynamic factor that favoured PLA. They calculated the polymer–polymer (PLA/PCL was 2.2 mN m^−1^) and polymer–filler (PLA/TiO_2_ was 7.5 and PCL/TiO_2_ was 15.6 mN m^−1^) interfacial tensions. The wetting coefficient was found to be 3.7, which satisfies the rule ω_α_ > 1, meaning the nanoparticles would preferentially localise in the PLA phase; however, in this study, more nanoparticles were seen to be localised at the interface. This behaviour could mean that PLA chains drove out the nanoparticles during mixing when their chains were aligning. To the best of our knowledge, we suggest that both thermodynamic and kinetic factors were favoured in this study due to the nanoparticles being localised in the PLA phase and at the interface between PLA and PCL.

**Figure 9:**
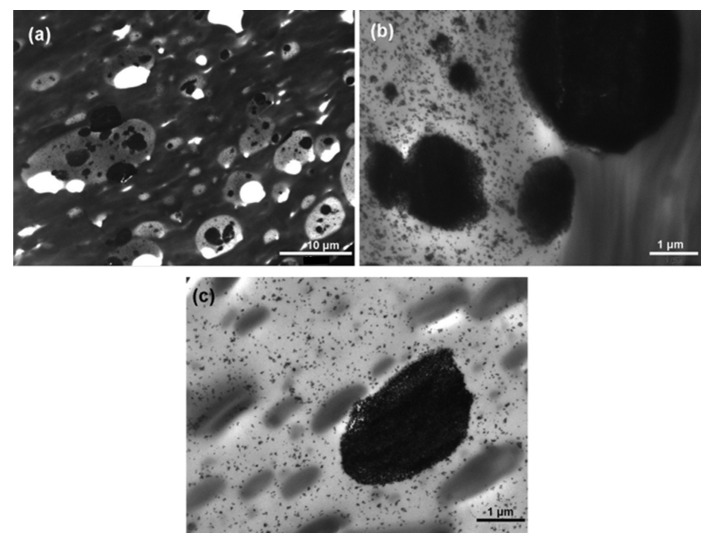
TEM images of 30/70/5 *w*/*w* PLA/PCL/TiO_2_ at (**a**) 1950× and (**b**) 13,500× magnification, and (**c**) 70/30/5 *w*/*w* PLA/PCL/TiO_2_ at 13,500× magnification [66]. (Reproduced with permission from ref. [66]. Copyright 2015, Elsevier).

Ostafinska et al. [113] made similar observations when evaluating the morphology of PLA/PCL/TiO_2_ nanocomposites by TEM. The authors reported that TiO_2_ was partly localised in the PLA matrix and partly at the PLA/PCL interface. However, a small agglomeration of TiO_2_ was observed with smaller sizes in the blends as observed by the study of Mofokeng et al. [119]. They attributed the smaller sizes of the nanoparticle agglomerates to the different melt mixing conditions used in this study. Furthermore, their results could mean that there is a strong affinity between TiO_2_ and PLA compared to TiO_2_ and PCL; hence, the nanoparticles are localised in the PLA phase and at the interface between the two phases. On the other hand, Mofokeng et al. [119] observed totally different behaviour when investigating the morphology of PLA/PCL/TiO_2_ nanocomposites with TEM. It was noted that most of the TiO_2_ nanoparticles were localised in the PLA matrix, meaning that there is a good affinity between PLA and TiO_2_, which was evident by well-dispersed nanoparticles in the PLA matrix (see Figure 9). However, the nanoparticles had several small and large aggregates present in the matrix. There was also no clear indication of TiO_2_ nanoparticles present at the interfaces between PLA and PCL. The authors explained their observations by surface properties, which indicated that the interfacial tension between PLA and TiO_2_ was very low (2.0 mN m^−1^), which explains why TiO_2_ was localised in the PLA matrix instead of the PCL phase because the interfacial tension between PCL and TiO_2_ was quite high (6.9 mN m^−1^). These results were further explained by the calculated wetting coefficient (ω_α_) value, obtained as 2.9 mN m^−1^, which is greater than 1. Based on the value calculated by Young’s relation, the preferential location of TiO_2_ nanoparticles should be in the PLA matrix, which agrees with the above discussion that TiO_2_ nanoparticles were localised in the PLA matrix.

More information on how fillers affect the morphology of the polymer blend is tabulated in Table 5. The focus of this table is only on the key points of the morphologies in the selected studies. It is noticeable from different studies’ perspectives that PLA/PCL are thermodynamically immiscible, and the incorporation of a third component (filler) could improve the adhesion between the PLA and PCL phases. However, the fillers are seemingly more efficient at lower contents, while at higher contents they agglomerate, forming defect sites that involve voids and cracks. These aggregates will disrupt the polymer blend’s morphology, thus resulting in a potential loss of desired mechanical performance. Miscibility in polymer blends is a key factor because it determines how the polymer components perform in the blend. Thermal analysis techniques, specifically differential scanning calorimetry (DSC) and dynamic mechanical analysis (DMA), are used to gauge miscibility in polymer blends, while thermogravimetric analysis (TGA) assesses the thermal stability of these polymers. In the section that follows, thermal analysis of pure polymers, and their blends and blend composites is evaluated and correlated with the polymer morphologies of the PLA/PCL blends and PLA/PCL blend micro-nanocomposites.

## 4. Thermal and Thermomechanical Properties

Thermal analysis is a group of techniques that measure a material’s physical properties when subjected to a controlled temperature. Techniques like differential scanning calorimetry (DSC), dynamic mechanical analysis (DMA) and thermogravimetric analysis (TGA) play a massive role in determining how polymers, and their blends and micro-/nanocomposites, behave when exposed to heat [121,122]. DSC measures how materials behave and respond when heated and cooled under certain or various conditions. This instrument can yield plenty of valuable data about a material’s properties, such as melting temperature (T_m_), glass transition temperature (T_g_), and crystallisation temperature (T_c_). It can also evaluate parameters such as the enthalpy of melting (ΔH_m_), cold crystallisation temperature (T_cc_), and enthalpy of crystallisation (ΔH_c_) of the material. Furthermore, the results of DSC depend upon the sample mass (normally 5–10 mg) of the tested material, and the heating and cooling rates [123,124]. DMA can be used to evaluate the glass transition temperature (T_g_) by assessing the storage (E′) and loss (E″) moduli of the polymers and polymer blends. In addition, T_g_ can also be identified from the peak in the tan δ (E″/E′) curve [66,104]. On the other hand, TGA measures the thermal stability of the material. With this instrument, the mass loss of the material is measured over time with respect to changes in temperature. It can also confirm phase separation through degradation temperatures, especially in multi-component systems [124]. Section 4.1 discusses the melting and crystallisation of the polymers and their blend and/or blend micro-nanocomposites, while Section 4.2 examines the dynamic mechanical properties of the polymers, polymer blends, and blend micro-nanocomposites. Lastly, Section 4.3 evaluates the thermal stability of the polymers and their blends and/or blend micro-nanocomposites.

### 4.1. Melting and Crystallisation of Pure PLA, PCL, Their Blends, and Blend Micro-Nanocomposites

**Figure 10:**
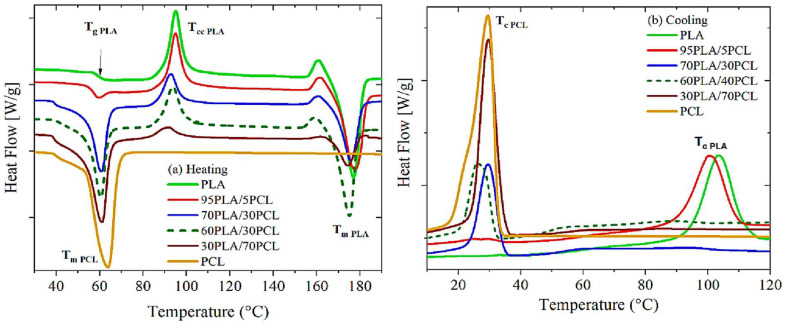
DSC thermograms of (**a**) heating and (**b**) cooling curves of pure PLA, PCL, and their blends [127]. (Open access from ref. [127]. Copyright 2025, MDPI).

The melting and crystallisation of polymers gives insight into their thermal behaviour. In this context, Patricio and co-workers [125] investigated the thermal behaviour of PLA/PCL blends produced by solvent casting. This work investigated 70/30 and 50/50 *w*/*w* PLA/PCL blends to look at the miscibility of the two polymers using DSC. It was observed that the melting temperature of neat PCL was 56.9 °C, and that of neat PLA was 153.3 °C, with a glass transition temperature of 58.5 °C. However, two separate melting peaks were observed in the blend, whereby the first endothermic peak was around 56 °C, which correlates to the PCL melting temperature, and the second peak around 150 °C, which is ascribed to the PLA melting temperature. However, the glass transition in the blends could not be observed because it coexists with the melting temperature of PCL. The fact that the PLA/PCL blends showed two separate melting peaks indicates that PLA and PCL are immiscible. Noroozi et al. [126] studied PCL/PLA blends prepared by the solution casting method. Two independent melting peaks were observed in the PLA/PCL blends, indicating immiscibility between PLA and PCL. This behaviour is consistent with what Patricio et al. [125] observed in their study. Matta et al. [100], when investigating biodegradable PLA/PCL polymeric blends, observed that the blends were immiscible, which was visible by the presence of two distinct melting peaks. Incorporating PCL reduced the degree of crystallinity and the melting temperature of PLA. It was also noted that PCL had no effect on the cold crystallisation temperature (T_cc_) of PLA, which was observed by non-shifting peaks of T_cc_ giving out similar values of T_cc_. Ivanov et al. [127] evaluated the phase separation and crystallinity of the PLA/PCL blends. The authors varied the PLA/PCL blend ratios and studied their thermal behaviour by DSC analysis, as seen in Figure 10. Three thermal transitions were observed with neat PLA, whereby the glass transition temperature was at 59.7 °C, the cold crystallisation peak at 95 °C, and the melting temperature at 177 °C. In contrast, one thermal transition was seen with neat PCL, with the melting peak at 63.8 °C. With PLA/PCL blends, two separate melting peaks were observed for all the blends. For example, at a 60/40 *w*/*w* blend, the peaks were seen at 59.9 and 175.1 °C, respectively. For the blends that show two distinct peaks, these are attributed to phase separation, which indicates that these two polymers are immiscible, as indicated by the authors of this study. Furthermore, the cold crystallisation peaks of PLA (T_cc_) shifted towards lower temperatures with increasing PCL content in the blends, from 95 to 91.7 °C. The cooling curves (b) showed that the crystallisation peaks of PLA were reduced with increasing PCL content. Moreover, the PCL content in the blends did not affect the crystallinity of PLA, except for the PLA/PCL (60/40 *w*/*w*) blend, which showed an increase in the crystallinity of PLA from 14 to 22%. The authors attributed this to a co-continuous morphology observed in SEM analysis, whereby each component in the blend contributes to the overall performance of the material.

Ferri et al. [128] evaluated the influence of PCL as a minor component on the thermal properties of the PLA/PCL blends. It was documented that neat PLA exhibited three thermal transitions: A glass transition temperature around 59 °C, cold crystallisation temperature at 100 °C, and melting temperature at about 168 °C. The addition of PCL did not affect the melting temperature of PLA, irrespective of the content used. This was seen by the melting temperatures of all the blends, that were around 168 °C. In addition, the glass transition temperature in the blends was not visible because it overlapped with the melting temperature of PCL. Furthermore, the degree of crystallinity of PLA was increased with increasing the PCL content from 16.5 to 17.2%. However, increasing the PCL content further reduced the degree of crystallinity of PLA to 14.1%. This shows that PCL disrupted the crystal growth of PLA by restricting its chain alignment to form an ordered crystalline structure because of the PCL excess in the blend. The authors concluded that PLA and PCL are immiscible because of the two main independent thermal transitions observed in the blend composition. It is worth noting that many studies in the literature on the PLA/PCL blends show that these two polymers are immiscible, characterised by the weak or poor interfacial adhesion between PLA and PCL [100,125,127,128]. Fillers in this case could be used to improve the poor interactions between PLA and PCL, and they can act as compatibilisers in the polymer blends. These fillers can interact with one or both polymers simultaneously and/or with the polymer interface and transfer stress to the polymer phases. Notably, they work efficiently when they are located at the interface, where they can improve poor interfacial tension and result in improved thermal and mechanical properties [50,60,66,67,68,96]. Rao et al. [57] investigated the effect of Montmorillonite (MMT) clay on the thermal properties of PLA/PCL blends. The 80/20 *w*/*w* blend was reinforced with 2, 4, and 6 wt.% of MMT. The addition of MMT nanoclay did not affect the glass transition temperature of the PLA phase in the blend. MMT also had minimal influence on the melting temperature of PLA in the blend. On the other hand, the melting and crystallisation enthalpies slightly increased with the increasing content of MMT. This behaviour could be attributed to MMT promoting crystal growth in the PLA/PCL blend by acting as a nucleating agent. Nanoclay at a higher content (6 wt.%) did not influence the degree of crystallinity of the nanocomposites, which could be due to agglomeration of MMT. At 4 wt.% content, nanoclay acted as a nucleating agent as it increased the degree of crystallinity from 31.5 to 33.2%. This could be ascribed to nanoclay having better interactions with the polymers than 6 wt.% MMT. More remarks are listed in Table 6, which discusses the thermal properties of the PLA/PCL blends and/or PLA/PCL blend composites. It is apparent from several studies that the glass transition temperature (T_g_) of the PLA/PCL blends is not quite visible in DSC analysis due to PCL’s melting temperature overlapping with PLA’s glass transition temperature. Thus, using another thermal technique, such as DMA, is crucial because the T_g_ of the polymer blends could be determined. In the section below, the usefulness of DMA is highlighted, and the observations of different studies are also correlated with the polymer morphology and DSC analysis of PLA/PCL blends and PLA/PCL blend micro-nanocomposites.

### 4.2. Dynamic Mechanical Analysis (DMA) of Pure PLA, PCL, Their Blends, and Polymer Blend Micro-Nanocomposites

**Figure 11:**
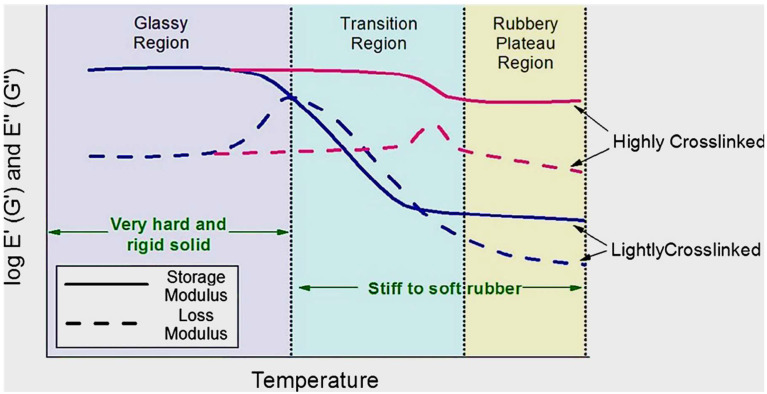
Illustrations of different regions of storage and loss modulus with respect to temperature [132]. (Reproduced with permission from ref. [132]. Copyright 2016, Elsevier).

This technique provides suitable information on the mechanical and rheological properties of materials. DMA is known to determine the glass transition temperature (T_g_), which plays a considerable role in determining the miscibility and/or phase separation of polymer blends. In addition, it can also be used to determine the phase transitions of the materials, stress relaxation, and effect of the fillers on dynamic properties (tan delta, storage, and loss modulus). Clear information about the material’s transition (from glassy to rubbery) is more visible in DMA compared to other thermal techniques (DSC) [66,104,131,132,133]. Figure 11 illustrates how materials transition in different regions as a function of temperature [132].

**Figure 12:**
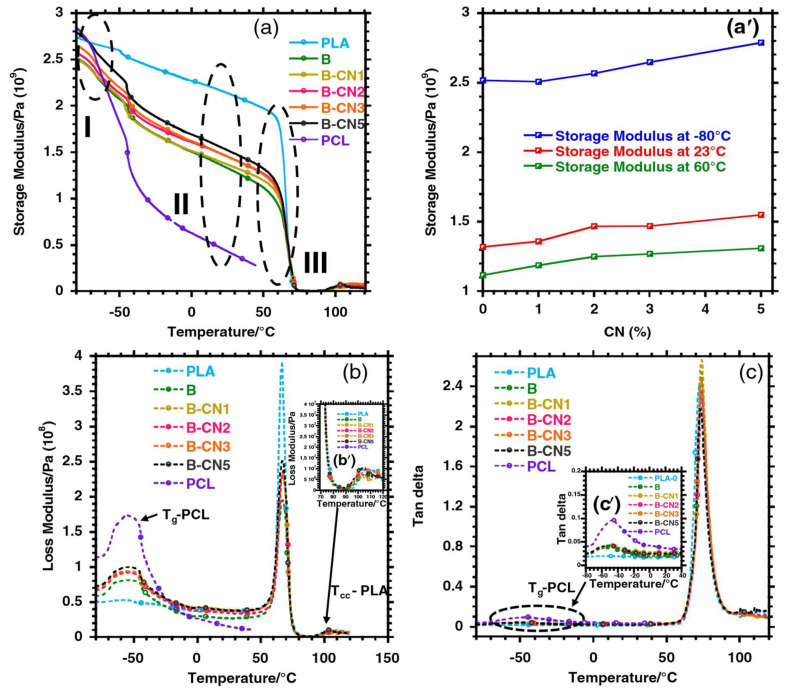
DMA curves of (**a**,**a′**) storage modulus, (**b**) loss modulus, and (**c**) tan delta of pure PLA, PCL, PLA/PCL blend noted B, and blend nanocomposites (B-CN1, B-CN2, B-CN3, B-CN5). The (**b′**,**c′**) are the insert figures of loss modulus and tan delta, respectively [60]. (Reproduced with permission from ref. [60]. Copyright 2020, John Wiley and Sons).

Botlhoko et al. [41] studied the dynamic mechanical properties of neat PLA, PCL, and their blends. The authors disclosed that the glass transition temperatures of pure PLA and PCL are noticeable at 67 and −42 °C, respectively. Two separable peaks of the glass transition were observed in the PLA/PCL blends, indicating that these two polymers are immiscible, which corresponds well with the immiscibility found in the morphologies of these blends in the SEM results. Matta et al. [100] investigated the viscoelastic properties of PLA and its blends (PLA90/PCL10, PLA80/PCL20, and PLA70/PCL30). The authors observed that increasing the PCL content in the blends decreased the storage modulus of the PLA. This behaviour was expected due to the flexibility of PCL, as the results showed that increasing its content reduced the stiffness of PLA. In contrast, the tan delta of PLA increased with increasing PCL content. An increase in the tan delta of PLA could mean that the damping ability was improved with the addition of PCL, and further improved with increasing content of PCL. A similar argument is supported by evidence from Yeh et al. [109] when investigating the properties of PLA/PCL blends. It was recorded that the storage moduli of PLA and PCL were 2700 MPa and 310 MPa at room temperature, respectively. Increasing the PCL content in the PLA blends reduced the storage modulus of PLA. On the other hand, there was an increase in the tan delta peaks of the blends in the presence of PCL. In terms of glass transition temperature, it was reported that increasing the PCL content in the blends decreases the T_g_ of the PLA phase from 64.3 to 58 °C. The dynamic mechanical analysis of the PLA/PCL blends reinforced with fillers and compatibilisers has been investigated. Fillers are known to influence the DMA of PLA/PCL blends by improving their viscoelastic properties, and that depends on the filler type, content, dispersion, and interfacial adhesion between the filler and the polymer blends [66,103,110,120,134]. In this instance, Mofokeng et al. [66] studied the dynamic mechanical analysis of PLA/PCL blends with titanium dioxide (TiO_2_) incorporated. The authors observed a very small change in the glass transition temperatures of PLA and PCL, confirming the complete immiscibility between the two polymers. They further observed a slight change in the glass transition temperature of PCL with the presence, as well as the increasing content, of TiO_2_ in the blend. The authors attributed this behaviour to the localisation of TiO_2_ nanoparticles into the PLA phase, which was observed in a TEM analysis. In addition, the glass transition temperature of PLA could not be determined because it occurs in the same melting temperature region as PCL. Jain et al. [134] explored the influence of micro-talc (1, 3, and 5 wt.%) on biodegradable PLA/PCL blends. According to the authors’ observation, the addition of PCL decreased the storage modulus of PLA from 3819 to 873 MPa. This could be caused by the softening of the PLA polymer, which resulted in the reduction of the storage modulus. However, the storage modulus of the PLA/PCL blend increased with increasing micro-talc, from 813 to 1377 MPa. Micro-talc is known to be a stiff filler, and its presence imparted rigidity to the blend. The glass transition temperature of the PLA/PCL/micro-talc composites decreased with increasing talc content. Furthermore, there was a reduction in the tan delta peak with increasing talc content, which could be ascribed to a restriction of the segmental mobility of PLA/PCL/micro-talc composites. Motloung et al. [60] studied the effect of 1, 2, 3, and 5 wt.% contents of cellulose nanocrystals (CN) on a biodegradable 70/30 *w*/*w* PLA/PCL blend, using DMA. The authors discussed the storage moduli of all the samples in three different regions, labelled I, II, and III in Figure 12a. The difference between these transition phases was described as follows: the transition phase (I) is known as the glassy region (temperatures below T_g_ of PLA and PCL); phase (II) is the transition region (known as the region that occurs between PLA and PCL T_g_ normally at room temperature); and phase transition (III) shows the rubbery region (the T_g_ of PLA), and all the regions are shown in Figure 11 and Figure 12a. It was observed that PCL has the highest storage modulus in region (I). The authors attributed that to PCL being a semi-crystalline polymer, so it has a reinforcing effect on crystals in that region. The lowest storage modulus was seen with the blend, which was attributed to two factors: the plasticisation effect of PCL in the PLA blend and the droplet–matrix morphology of the blend examined by SEM analysis. It was further observed that when CN was incorporated in the blend, there was an increase in the storage modulus, indicating that rigid CN nanoparticles acted as a reinforcing agent. Furthermore, CN had no influence on PLA’s glass transition, as Figure 12a (region III) shows, and this was ascribed to CN not localising in the PLA phase. Looking also at the tan delta (Figure 12c, at region I), there was no effect of CN on the T_g_ of PCL, even though CN was reportedly located in this phase. The authors attributed this behaviour to the highly hydrophilic nature of CN and the hydrophobic nature of PCL. Their interaction will therefore be poor, which was expected by the authors; hence, no significant change was observed in the T_g_ of PCL. It is evident that the dynamic mechanical properties of polymer blends and composites are crucial in assessing the mechanical performance of the material under thermal conditions. Section 4.3 provides a comprehensive understanding of the thermal stability/degradation of pure PLA, PCL and their blends and composites.

### 4.3. Thermal Degradation of Pure PLA, PCL, Their Blends, and Blend Composites

**Figure 13:**
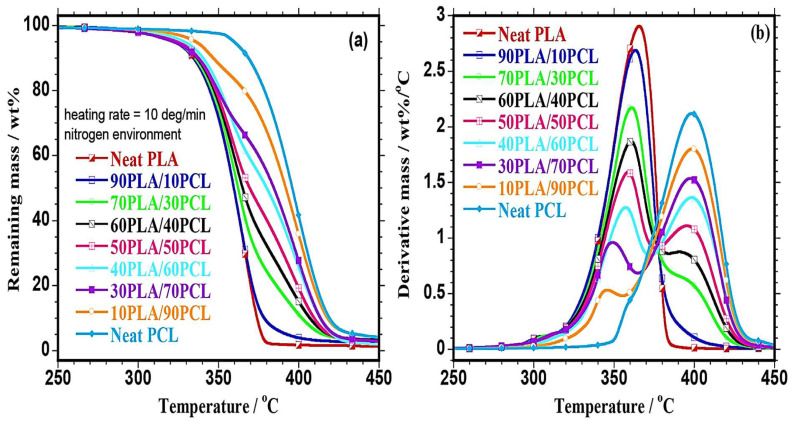
TGA (**a**) and DTG (**b**) curves of neat PLA, PCL, and PLA/PCL blend with varying blend compositions [41]. (Reproduced with permission from ref. [41]. Copyright 2018, Elsevier).

Thermal stability/degradation solely depends on the degree of crystallinity, chemical structure, and molecular weight of the materials [41,66,135]. Botlhoko et al. [41] evaluated the thermal stability of PLA/PCL blends with varying blend compositions (Figure 13). It was reported that both PLA and PCL showed single-step degradation, whereby the onset temperatures or T_10%_ of PLA and PCL were 335.2 and 368.2 °C, respectively. The maximum degradation temperature of PLA was 364 °C, and that of PCL was 398 °C, which is depicted in Figure 13b. The results indicate that PCL is more thermally stable compared to PLA polymer due to its higher thermal degradation temperature. The degradation mechanism of PCL occurs via a two-step process, whereby the first step involves random chain scission and the second step is characterised by unzipping depolymerisation. This two-step process produces products like hexanoic acid and ε-caprolactone. PLA degradation occurs via a range of mechanisms, including hydrolytic, thermal, enzymatic, and microbial, to mention a few. Hydrolysis is a key factor in PLA degradation, which occurs via the ester linkages. This process can be accelerated by higher temperatures, as well as in the presence of water. PLA can degrade back into its monomer (lactic acid), and this monomer can further be broken down into water and carbon dioxide as byproducts [24,28,135,136,137]. Regarding the PLA/PCL blends, two degradation steps were observed: the maximum degradation temperatures of the 70/30 *w*/*w* PLA/PCL blend were at 361.06 and 380.5 °C, respectively. The two temperatures represent the respective degradation of PLA and PCL, confirming immiscibility between the two polymers, which the authors correlated to a phase-separated morphology on SEM analysis for all the blends. It is worth noting that the authors expected that incorporating higher thermally stable PCL into PLA would increase its thermal stability. However, that was not the case, because at 10% weight loss (T_10%_), the authors observed a slight reduction in the thermal degradation for 90/10 and 70/30 *w*/*w* PLA/PCL compositions as opposed to pure PLA. They attributed this behaviour to the PCL (dispersed phase in those above-mentioned compositions) forming droplets of different sizes in the PLA matrix. In contrast, different behaviour was observed for the blends that had higher PCL contents (60/40, 50/50, 40/60/, 30/70, and 10/90 *w*/*w*) whereby the thermal stability at T_10%_ was increased. On the other hand, the thermal stability of all the blends was increased at 50% weight loss (T_50_%) in relation to pure PLA. This behaviour was ascribed to the higher PCL content in the blend and its high thermal stability, meaning it improved PLA’s thermal stability, hence the increase in PLA stability was observed. In other words, at this weight composition (50/50 *w*/*w*), the blend inherited the high thermal stability of PCL, resulting in the blend shifting closer to pure PCL’s thermal stability. Mofokeng et al. [66], Matumba et al. [135], and Bouakaz et al. [136] all observed similar behaviour of neat PLA and PCL, in that they both degrade in a single-step degradation, whereas the PLA/PCL blend degrades in a two-step degradation, which confirms phase-separated systems.

**Figure 14:**
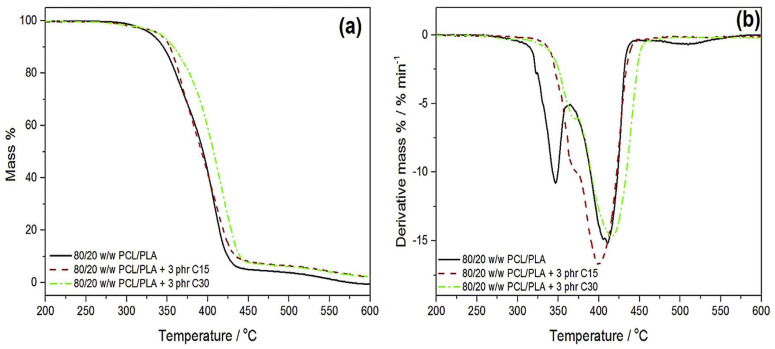
TGA (**a**) and DTG (**b**) curves of the PLA/PCL blend and the composites with different nanoclays (C15A and C30A) [137]. (Reproduced with permission from ref. [137]. Copyright 2016, Elsevier).

Chomachayi and co-workers [61] studied the thermal behaviour and performance of the PLA/PCL blend and silk fibroin nanoparticles (SFNPs). The authors stated that both PLA and PCL degraded in a single step, whereby PLA degraded at a maximum temperature of 374.3 °C and PCL at 412 °C, respectively. This behaviour confirms that PLA has lower thermal stability in comparison to PCL, as already stated. With the blends, the findings are the same as reported by Mofokeng et al. [66], Matumba et al. [135], and Bouakaz et al. [136] in the prior section. The authors reported that adding SFNP to the PLA/PCL blend reduced the onset degradation temperatures (T_5%_ and T_10%_) of the blend. SFNPs reduced the degradation temperature of the blend, and this may be due to the presence of acidic groups (aspartic acid) in it. These acid groups catalyse the degradation of polymers by enabling enzymatic attack on hydrophilic domains. However, there was an increase in the degradation temperature of the PLA/PCL blend at T_95%_ in the presence of SFNPs. This behaviour was attributed to the high thermal stability of SFNPs, which acted as a heat barrier by protecting PLA and PCL chains from thermal degradation at higher temperatures. Though the authors did not mention it, the SFNPs seemed to have had an autocatalytic effect on the thermal stability of the blend, accelerating the degradation at lower temperatures and retarding it at higher temperatures. Mofokeng et al. [137] analysed the influence of two different clays (Cloisite 15A and Cloisite 30A) on the thermal stability of the PCL/PLA blend (80/20 *w*/*w*). The study revealed a two-step degradation of the PCL/PLA blend; however, the steps were not that pronounced, as shown in Figure 14a,b. In the presence of both Cloisites (C15A and C30A), the degradation step of PLA shifted to higher temperatures, almost merging with that of PCL. As seen from Figure 14a, both the onset and maximum degradation temperatures of the 80/20 *w*/*w* PCL/PLA blend were delayed/shifted to higher temperatures with Cl5A and C30A. It could also be implied that both C15A and C30A were more thermally stable than the PCL/PLA blend. In addition, the derivative curves in Figure 14b confirm the two-step degradation of the neat PCL/PLA blend (80/20 *w*/*w*), which is more pronounced than in Figure 14a. The first degradation peak is at 346 °C, and the second degradation peak is at 411 °C. The first step is ascribed to PLA degradation, whereas the second step is attributed to PCL degradation. Regarding both the composites with C15A and C30A, it is quite clear that the clays interacted with PCL and PLA, as a result compatibilising the blend and possibly improving the miscibility. This behaviour was seen by the two-step degradation almost merging into one-step degradation with both Cloisites. Based on this information, it can be ascertained that the thermal stability of the most studied PLA/PCL blends could be swayed by the blend ratio and the dispersion of the blend components. In most cases, two-step degradation is observed in the PLA/PCL blends, where each step corresponds to the degradation temperature of the respective polymer. The incorporation of fillers was found to improve the thermal stability of PLA/PCL blends in most of the studies. As with the thermal degradation and stability, the mechanical properties of the materials are also important if we wish to develop a holistic understanding of these materials, to allow for their effective applications. The mechanical properties of polymer blend composites are discussed in the following section.

## 5. Mechanical Properties

The mechanical performance of the materials is very important because it reflects how polymers, polymer blends, and polymer/blend composites perform under different conditions (temperature, load, and rate). Mechanical analysis is a technique that can evaluate the mechanical performance of polymer blends and micro-nanocomposites. This technique provides suitable information, like elasticity, tensile strength, hardness, and toughness, for the material’s practical use in various applications. Examining the mechanical properties of the polymers and their blends and blend composites is essential for determining their usefulness or suitability in practical applications.

It has been stated in the literature that factors such as processing methods, blend ratios, content of micro/nanofillers, crystallinity of polymers, synergistic effect of fillers, phase interactions (filler–filler, filler–polymers), and surface modification of fillers have an influence on the overall mechanical properties of PLA/PCL blends and/or PLA/PCL blend micro/nanocomposites [120,138,139,140]. This section discusses in detail the influence of some of the above-mentioned factors.

### 5.1. Effect of Varying Blend Composition on PLA/PCL Blends’ Mechanical Properties

**Figure 15:**
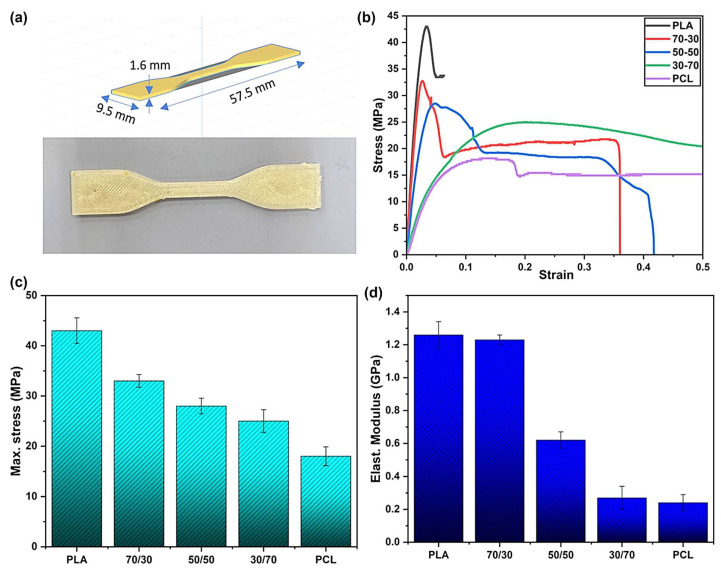
Mechanical properties showing (**a**) 3D-printed specimen with its dimensions, (**b**) stress vs. strain curve of PLA, PCL and their blends (70/30, 50/50, and 30/70 *w*/*w*), (**c**) Max stress (ultimate tensile strength), and (**d**) Elastic modulus of the various samples [141]. (Open access from ref. [141]. Copyright 2025, De Gruyter Brill).

Various studies indicate that varying the PLA and PCL content could significantly enhance/reduce the mechanical properties of the PLA/PCL blends [56,128,129,139,141]. Qiu et al. [139], for example, evaluated the effect of PCL content on the mechanical properties of PLA/PCL blends. The authors highlighted a reduction in the tensile strength of the PLA/PCL blends with increasing PCL content, and they attributed that to the reduction in the degree of crystallinity of the blend as PCL is added. On the other hand, increasing the PCL content increased both the impact strength and elongation at break of the PLA/PCL blends. This ductile behaviour is linked to the flexibility property of PCL, which effectively improved the toughness of PLA. Ferri et al. [128], in their study, reported that all the investigated PLA/PCL blends’ tensile strengths and tensile moduli were reduced by increasing the PCL content. This behaviour was expected due to the good elastic property of PCL, which allowed the PLA blends to deform without fracturing, which confirms the findings outlined above [139]. It is also noted that not only the addition of PCL but also its amount/content influences PLA’s mechanical properties. The authors stated that incorporating 22.5 wt.% PCL content tremendously increased the elongation at break, but a further increase in PCL content to 30 wt.% resulted in a reduction in the elongation at break. This could be due to phase separation and poor interfacial adhesion between PLA and PCL, as such an increase in PCL content could change phase morphology by worsening phase separation, which is responsible for premature failure during stretching. This shows that it is important for researchers from time to time to conduct studies in line with the optimal contents of the components to effect necessary changes. As it stands, it is not clear at which point between 22.5 and 30 wt.% content the PCL stopped improving PLA’s elongation at break. Kalva et al. [141] prepared 100/0, 70/30, 50/50, 30/70, and 0/100 *w*/*w* PLA/PCL blend compositions, and used them as filaments to manufacture samples using a 3D printer. The authors then investigated the mechanical properties of the 3D-printed PLA/PCL blends, and their tensile tests are reviewed in Figure 15. It can be seen from the stress–strain curves that increasing the PCL content in the blends reduced the breaking stress (tensile strength) of the PLA polymer (Figure 15c). These results show that PCL enhanced the ability of the blends to deform without breaking easily, unlike pure PLA. PCL significantly impacted the elongation at break of PLA by increasing it from 5 to above 50%, and this can be attributed to the flexibility property of PCL in the blends. The elastic modulus of PLA was also reduced with increasing PCL content, which is illustrated in Figure 15d. This behaviour was expected because of the high flexibility of PCL, which reduced the stiffness of PLA.

Research by Wei et al. [56] supports the findings of Qiu et al. [139] and Ferri et al. [128] that increasing the content of PCL in blends reduces tensile strength while increasing elongation at break. The elongation at break of PLA is very low, about 16%, indicating its brittleness. Adding 10 wt.% PCL content into PLA led to a tremendous increase in elongation at break, about 138%. However, increasing the PCL content further resulted in a decrease in elongation at break, which could be due to PCL becoming the continuous phase and PLA becoming the dispersed phase. This implies that the dispersed PLA phase could act as rigid inclusions, which will promote weak points and initiate early failure due to hindrances to stress transfer being generated in the blends. Urquijo and co-workers [142] observed that the incorporation of PCL (10 wt.%) proved effective as it increased the elongation at break of PLA to 140%. The effectiveness of PCL at lower contents was due to its domains being well dispersed in the continuous PLA phase, thus significantly improving the ductility of the blend. Nonetheless, higher PCL contents proved ineffective because the elongation at break remained unchanged. This behaviour could be related to weak interfacial adhesion between PLA and PCL at higher PCL contents. Chen et al. [129] found that increasing the PCL content in the PLA/PCL blends reduces the tensile strength of the materials from 12 to 6.7 MPa. Furthermore, the elongation at break of PLA was significantly increased, from 162.265 to 175.353%. These findings are consistent with the observations of the previous studies on PLA/PCL blends [56,128,139].

### 5.2. Effect of Micro/Nanofillers on Mechanical Properties of Polymer Blend Micro-Nanocomposites

**Figure 16:**
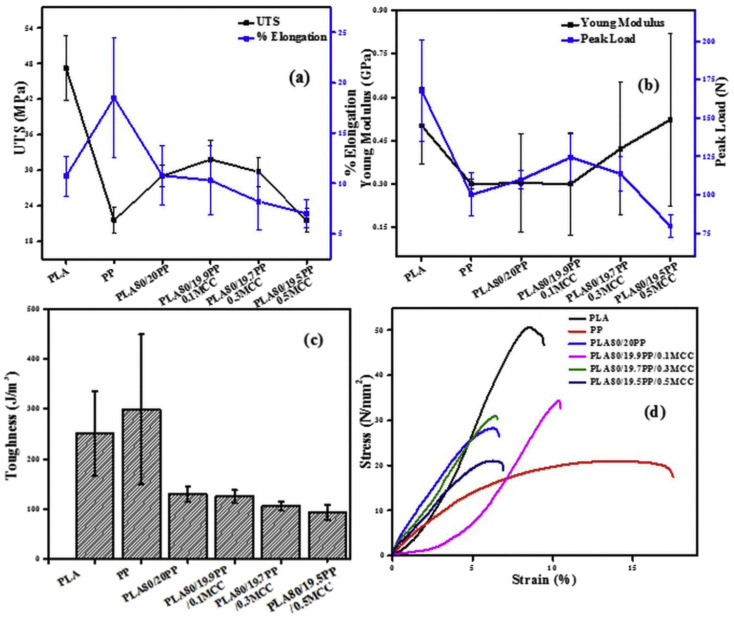
(**a**) Ultimate tensile strength, (**b**) Elongation and Young’s modulus, (**c**) Toughness, and (**d**) stress vs. strain curve of PLA, PP, PLA80/20PP and its biocomposites [138]. (Reproduced with permission from ref. [138]. Copyright 2020, Elsevier).

Micro/nanofillers have been previously used as reinforcing materials to enhance the mechanical properties (tensile strength, elongation at break, toughness, and Young’s modulus) of polymer blends because of their strong interfacial bonding with polymers. These fillers can offer high surface area and stress transfer to polymer blends, which results in outstanding mechanical performance [57,87,120,143,144]. Proper dispersion of fillers within the polymer blends is essential because it could provide excellent mechanical reinforcement, and this depends on the filler localisation. It is also worth noting that adding excessive amounts of fillers within polymer blends often leads to a significant increase in the system’s viscosity, resulting in poor processability of the materials [104,145,146,147]. Poljacek et al. [120] reported their findings on the effect of nano-silica and poly(e-caprolactone) on the mechanical properties of poly (lactic acid)-containing blends. It was stated that the elastic moduli of all the blends increased with the addition of 1 and 3 wt.% nano-SiO_2_. The authors expressed that this could mean silica acted as a reinforcing agent, hence the increased elastic modulus of the materials. Silica nanoparticles slightly increased the tensile strength of only the 50/50 and 60/40 *w*/*w* PLA/PCL blends, which could mean the interfacial bonding between the materials was excellent, according to the authors. Rao et al. [57] investigated the enhancement of the mechanical properties of an 80/20 *w*/*w* PLA/PCL blend with MMT nanoclay addition. It was revealed that the tensile strength and modulus of the PLA/PCL blend increased with the addition of clay. The highest tensile strength and modulus were observed at 4 wt.% clay content. Furthermore, at 6 wt.% clay content, there was a decrease in the tensile strength and modulus of the PLA/PCL blend, possibly due to agglomeration of clay in the composites. These agglomerates are said to prevent PLA and PCL chains from interacting with the clay; in other words, in this system, there were particle-to-particle interactions instead of polymer–particle–polymer interactions. Haghgoo et al. [147] explored the effect of cellulose nanocrystals (CNCs) on the mechanical performance of a PLA/PCL blend. The authors incorporated 0.25, 0.5, and 1 wt.% CNC content into an 80/20 *w*/*w* PLA/PCL blend, and studied the mechanical properties. It was reported that the addition of CNCs improved both the tensile strength and modulus of the PLA/PCL blend. The authors attributed this improvement to the strong compatibility effect of CNCs seen in their SEM analysis, which showed a decrease in the droplet sizes of the PCL component in the blend. It is further pointed out that the elongation at break was reduced with increasing CNC content. The reason associated with this reduction was the stiffness of the CNCs, which restricted the polymer chains’ mobility, resulting in increased brittleness of the composites. Bhasney et al. [138] incorporated MCC to improve the mechanical properties of 80/20 *w*/*w* PLA/PP blends. Upon the addition of MCC to the PLA/PP blend, the authors observed an increase in the ultimate tensile strength (UTS) with 0.1 wt.% MCC content with respect to the neat blend, but a further increase to 0.5 wt.% resulted in a reduction in the UTS of the blend composites, as illustrated in Figure 16. The elongation at break of the blend decreased with increasing MCC fibre content. These results could mean that there was a weak stress transfer between the MCC fibre and the blend; hence, lower tensile strength was observed. The authors further observed a decrease in the Young’s modulus of the composites containing 0.1 wt.% MCC content. However, with the addition of 0.3 and 0.5 wt.% MCC to the PLA/PP blend, there was an improvement in the Young’s modulus of the PLA/PP blend. The authors suggested that the improvement in the Young’s modulus could be due to the fibre alignment and aspect ratio of the MCC fibres in the blend. For instance, there will be an increase in Young’s modulus if the MCC fibres are oriented parallel to the direction of the stress transfer, thus transferring stress effectively between the blend and the MCC fibres. Aspect ratio plays a role in this sense: a high-aspect-ratio fibre gives excellent reinforcement, while a low-aspect-ratio fibre provides poor reinforcement due to pull-out of fibres.

### 5.3. Effect of Hybrid Fillers and/or Fillers with a Compatibiliser on the Mechanical Properties of PLA/PCL Blends

**Figure 17:**
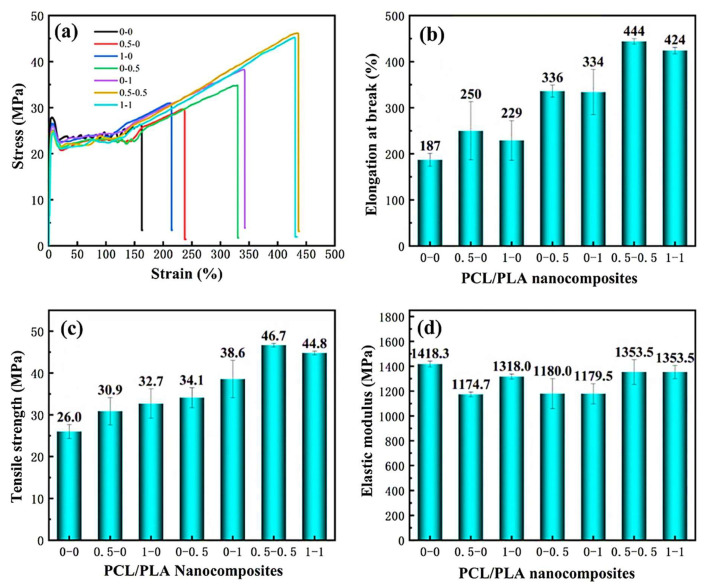
Mechanical properties of PCL/PLA nanocomposites: (**a**) stress–strain curves, (**b**) elongation at break, (**c**) tensile strength, and (**d**) elastic modulus [130]. (Reproduced with permission from ref. [130]. Copyright 2020, Elsevier).

A hybrid filler refers to the combination of two or more types of fillers in the polymer component system. The combination of fillers is expected to outperform individual fillers by combining their properties in the blend system, which can provide superior mechanical properties. These fillers could complement each other by creating and forming an interpenetrating network that can provide better overall performance of the polymer blend micro-nanocomposites [130,148,149]. There is, however, limited work that has been produced exploring the effect of hybrid fillers and/or fillers with a compatibilizer on the mechanical properties of PLA/PCL blends [65,130,150]. Zhu et al. [130] inspected the effect of both multi-walled carbon nanotubes (CNTs) and montmorillonite (MMT) on the mechanical properties of PCL/PLA nanocomposites. Their investigation was on a 70/30 PCL/PLA blend with 0.5 and 1.0 wt.% of both CNTs and MMT. The stress–strain, tensile strength, elastic modulus, and elongation at break curves are represented in Figure 17. It was reported that the elongation at break and tensile strength of the PCL/PLA blend (187% and 26 MPa) increased with increasing contents of both CNTs and MMT in all the nanocomposites. The increase in elongation at break of the PCL/PLA blend was from 187 to 250 and 336%, with 0.5 wt.% of CNTs and MMT, respectively (Figure 17b). On the other hand, the tensile strength of the PCL/PLA blend was increased from 26 to 32.7 and 38.6 MPa with 1 wt.% CNTs and MMT, respectively (Figure 17c). The addition of MMT significantly improved the elongation at break and tensile strength of the PCL/PLA blend compared to CNTs. The authors attributed this observation to the position of MMT, which was located at the interface between the two phases. However, there was a slight reduction in the elastic modulus of the PCL/PLA blend with the addition of solely MMT and CNTs. The authors attributed this behaviour to a decrease in the crystallinity of the matrix (PCL), which was confirmed by the wide-angle X-ray diffraction (WAXD) results. The authors stated that when MMT and CNTs are loaded simultaneously, the elongation at break and tensile strength of the PCL/PLA blend are significantly improved compared to the sole addition of both fillers. Elongation was improved from 187 to 444 (0.5 wt.% CNTs and MMT) and 424% (1 wt.% CNTs and MMT), respectively. The tensile strength was improved from 26 to 46.7 and 44.8 MPa for the respective filler compositions stated above. The authors attributed the synergistic effect of MMT and CNTs to morphology observations, whereby the exfoliated MMT platelets improved interfacial adhesion between PCL and PLA, while CNTs formed an interpenetrating network in the blend [128].

Negaresh et al. [65], in their study, addressed the effect of adding a compatibiliser (glycidyl methacrylate (GMA)) and nanoparticles (nano-calcium carbonate (NCC)) into PLA/PCL blends. The authors found that GMA and NCC significantly impacted the mechanical properties of PLA/PCL blends. This behaviour was seen by GMA improving the elongation at break and impact strength of the PLA/PCL blends. At the same time, NCC improved the tensile modulus and impact strength. In one study by Ye et al. [150], the mechanical properties of the PLA/PCL/microcrystalline cellulose (MCC) compatibilised with maleic anhydride-grafted PLA (PLAma) were examined. According to the authors, the inclusion of PCL led to a remarkable improvement in the elongation at break of PLA, but a reduction in the strength and elastic modulus, as seen from Table 7. This improvement is related to the rubbery state of PCL, and the reduction was attributed to the elastic modulus and poor strength of PCL. Incorporating microcrystalline cellulose (MMC) content, as well as increasing it, in the PLA/PCL blend resulted in a reduction in strength and elongation at break. The elastic modulus of the composites was, however, increased with increasing MMC content. The reason for this occurrence is that MCC possesses rigid properties; as such, increasing its content provides rigidity to the composites, which increases elastic modulus. After incorporating a compatibiliser (PLAma) in the composites of PLA/PCL/5MCC, there was an increase in the strength, elongation at break, and elastic modulus of the PLA/PLAma/5MCC/PCL composites. These observations could be due to the compatibiliser improving the interfacial adhesion between the components in the composites.

### 5.4. Effect of Surface Modification of Fillers on the Mechanical Properties of the PLA/PCL Blend Micro-Nanocomposites

**Figure 18:**
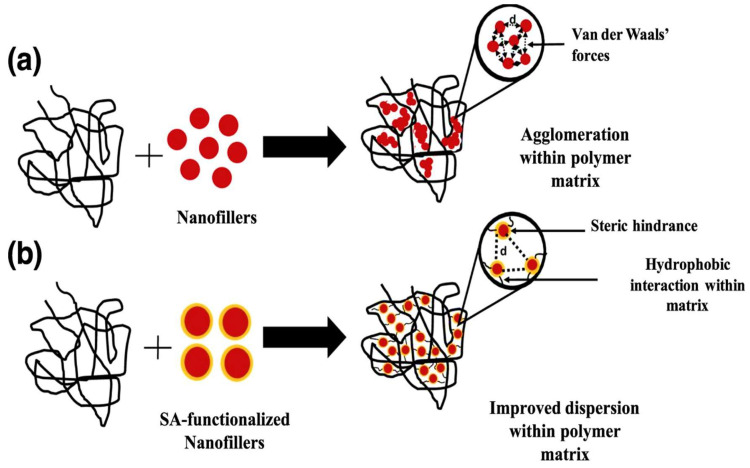
Illustration of the effect of non-functionalised (**a**) and surface functionalised (**b**) nanofillers on polymer nanocomposites, and d is the inter-particulate distance, showing increasing and improving dispersion [140]. (Open access from ref. [140]. Copyright 2018, Springer Nature).

There are many limitations concerning the fillers in polymer blends, which could be associated with their dispersion, agglomeration, and hydrophilic nature. It is well known that unmodified fillers are effective to some extent. However, adding excessive contents into the polymer matrices and/or blends can hinder their dispersion and cause agglomeration. This behaviour then leads to poor mechanical performance of the composites/blend composites [151]. Hydrophilic fillers are also said to cause a decline in the mechanical properties of PLA/PCL blends because both PLA and PCL are reportedly hydrophobic in nature, though some reports show otherwise with contact angle measurements. For example, hydrophilic fillers tend to agglomerate with hydrophobic polymers, due to poor interfacial adhesion between polar and non-polar components. This is reported to then lead to a weak stress transfer at the interface, resulting in poor mechanical properties. Surface modification in this case could be utilised to enhance the dispersion and interfacial adhesion between the polymers and the fillers. This would result in a reduction of the agglomeration of the fillers in the polymer matrix and/or blend composites and possibly improve the mechanical properties [140,152]. Ang et al. [138], in their study, evaluated the effect of unfunctionalised barium sulfate (BaSO_4_) nanofillers and steric acid (SA)-functionalised BaSO_4_ nanofillers on the mechanical properties of poly-l-lactide (PLLA) composites. The authors used the image in Figure 18 to explain the behaviour of functionalised and unfunctionalised fillers. In this image, they showed that unfunctionalised nanofillers usually form agglomerates within the polymer matrix due to Van der Waals forces causing particle-to-particle interactions. These aggregates could weaken the interactions between the filler and the polymer (PLLA), resulting in poor mechanical properties. However, with functionalised nanofillers, the surface modification results in the separation of particles due to steric hindrance, improving the dispersion. With the nanofillers dispersed within the polymer matrix, the stress will be effectively transferred by the fillers, thereby improving mechanical properties [140].

Nethula and co-workers [153] evaluated the use of silica-coated TiO_2_ (S-TiO_2_) nanoparticles incorporated into a polylactic acid (PLA)/thermoplastic polyurethane (TPU) composite. The contents of S-TiO_2_ added into the 70/30 *w*/*w* PLA/TPU blend were 0.5, 1.0, 1.5, and 2.0 wt.%. As stated by the authors, the tensile strength was significantly increased from 36 MPa to 46 MPa with the addition of 2 wt.% of S-TiO_2_. This can be ascribed to a homogeneous distribution/dispersion of the filler, thus increasing the interfacial adhesion between PLA and TPU, and resulting in efficient stress transfer. In summary, the addition of organic and inorganic fillers is found to improve the mechanical performance of PLA/PCL composites, especially for applications in personal hygiene (e.g., diapers, sanitary towels). The mechanical performance of the composite system could also have a significant impact on the barrier properties of the composites. This could be due to the dispersion and alignment of fillers in the polymer blends; as such, improved mechanical properties could mean that the barrier properties will be improved. The following section assesses the effects of fillers on barrier properties within polymer blends.

## 6. Barrier Properties of Neat PLA, PCL, PLA/PCL Blends, and PLA/PCL/Micro-Nanocomposites

Barrier properties of polymers can be referred to as the ability of a material to withstand or limit the permeation of substances like liquids or gases that go through the polymer material, to preserve the material’s quality. These properties can be affected by different parameters such as the degree of crystallinity of polymers, chemical compositions of the components, and the presence of fillers (type and shape) in the polymer system [61,154,155]. The water vapour permeability (WVP) test is a crucial technique for measuring water vapour passing through a material, especially in packaging and other protective applications (in our case, personal hygiene products) [61,154,156]. In the literature, it is stated that blends and composites’ (polymer–polymer, and polymer–micro/nanofiller) barrier properties may vary because they depend on the nature of the components, and also on the final morphology [108]. Zhang et al. [157] investigated the effect of other biodegradable polymers on PLA-containing blends. In this study, the authors focused on improving the water-resistance properties of PLA, which was achieved by lowering the water vapour permeability (WVP). It was reported that the addition of PCL into PLA reduced the WVP from 15.0 to 3.1 × 10^−14^ g·cm/cm^2^·s·Pa. Other biodegradable polymers were used in this study, as listed in Table 8. However, the focus here is on the PLA/PCL blend, which is the blend studied in this review.

The results in Table 8 indicate that all the polymers blended with PLA successfully reduced the WVP in the resulting blends, compared to that of neat PLA. The authors found these observations particularly beneficial for the preservation of strawberries, and stated that a smaller WVP value is conducive to maintaining product freshness. However, it is worth acknowledging that the findings in the literature on the WVP of PLA/PCL blends are not consistent. The effect of PCL on WVP can vary significantly depending on the blend ratios, the degree of phase compatibility between PLA and PCL, and the different grades of these polymers. For instance, the study of Moraczewski et al. [158] on PLA/PCL polymer material for food packaging reported opposite trends. The authors reported that the addition of a lower PCL content (15 wt.%) did not have an impact on the WVP of the PLA polymer, as it remained unchanged at 0.71 × 10^−14^ g·cm/cm^2^·s·Pa. However, increasing the PCL content in the blend further (30 wt.%) led to a significant change in WVP, which reached 2.09 × 10^−14^ g·cm/cm^2^·s·Pa. This behaviour was attributed to phase separation and increased material flexibility, which allowed the transport of water vapour. Chomachayi et al. [61] studied the barrier properties of PLA/PCL blends in the presence of silk fibroin particles (SFNPs). They selected a 70/30 *w*/*w* PLA/PCL blend as their optimised blend and incorporated 1 wt.% of SFNP content to perform a water vapour permeability (WVP) test. Their results showed that the PLA/PCL blend had higher permeability than neat PLA. The reason could be related to a phase-separated morphology, and incompatibility, which could have created a pathway for the transport of water vapour molecules, according to the authors. However, in the presence of 1 wt.% SFNPs, the results showed a remarkable reduction in the WVP value, by 16%. This behaviour was ascribed to the hydrophobic nature of SFNPs and their good dispersion in the PLA/PCL blend, which created a more tortuous path that restricted the transport pathways of small molecules like water and gases. Based on the observations of the WVP test and other techniques like SEM, DSC, TGA, and mechanical properties, which are not discussed in this section, the authors concluded that PLA/PCL-SFNP nanocomposites have excellent capability for use in food packaging applications. It is worth mentioning that both low and high values of WVP could be essential for the overall performance of polymer blend composites, depending on the desired application. For instance, a lower WVP is beneficial for applications in packaging and building materials, whereas a higher WVP is essential for medical textiles and clothing. The section below gives an insight into different applications based on PLA and PCL in the presence of micro- or nanofillers.

## 7. Applications Based on PLA/PCL/Micro-Nanofiller Composites

Biodegradable polymers (PLA and PCL) filled with hybrid fillers (micro-nanofillers) represent a novel approach that has potential for improving mechanical, barrier, thermal, toxicity, and other properties. Despite this, there is a gap in polymer blend composites, specifically PLA/PCL/micro-nanofillers, with regard to hygiene and health-related applications (surgical masks, diapers, wipes, and sanitary pads) being reviewed and studied. To the best of our knowledge, there are only limited studies that use the exact combination of these components. Presently, academic researchers and manufacturing industries have taken an initiative to capitalise on the development of new biodegradable blend micro-nanocomposites for intended or suitable applications [159]. The widespread use of biodegradable polymers has increased in both industrial and academic research in order to lessen the volume of petroleum-based products currently being disposed of in landfills [19]. To give an example, Alaswad et al. [5] provided an overview of the probable biodegradable polymers that could be used in medical and hygiene applications to replace synthetic polymers. The findings of this work are summarised in Table 9, showing the medical products, materials applied, and probable replacements of petroleum-based polymers. From this table, it can be deduced that biodegradable polymers, particularly PLA, have exceptional properties and the potential to be used in various disposable medical and hygiene products to alleviate plastic pollution.

**Figure 19:**
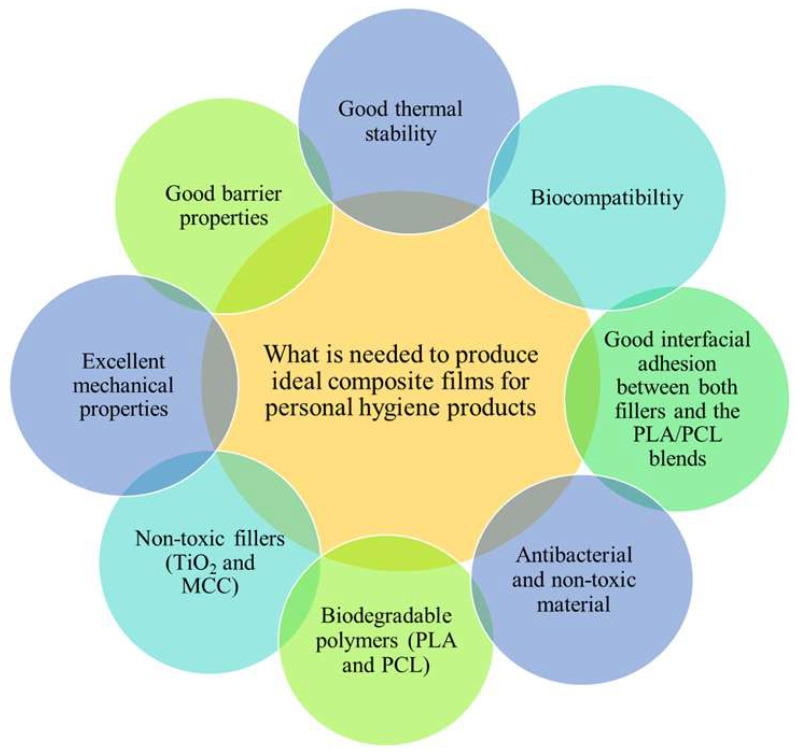
Requirements to produce composite films for use in personal hygiene products.

Biodegradable polymers have been studied by numerous researchers for different biomedical and packaging applications [113,160,161,162]. For example, Sundar et al. [160] evaluated biodegradable PLA/PCL blends for packaging applications. In this study, petroleum-based kraft paper was replaced with biodegradable PLA/PCL kraft paper. Various tests, like water vapour transmission rate (WVTR), heat seal strength, and mechanical properties, to name a few, were performed to evaluate the kraft paper’s suitability for packaging applications. The optimal composition chosen in this study was 90/10 *w*/*w* of the PLA/PCL blend. The WVTR test showed that the PLA WVTR value was reduced with the incorporation of PCL (10%), from 628 to 615 g·m^−2^ × 24 h. In addition, the coated biodegradable PLA/PCL kraft paper had a lower WVTR value (615 g·m^−2^ × 24 h) in comparison to virgin kraft paper (836 g·m^−2^ × 24 h). The PLA/PCL blend also exhibited good heat seal strength, with a value of 76.6 kPa. Regarding mechanical properties, the tensile strength and elongation at break were increased with 10% PCL content. The authors concluded that blending PLA with PCL at a lower content (10%) improved the properties of the PLA polymer. These results showed that this blend could be exploited in industrial products owing to good barriers, heat seal strength, and mechanical properties. In another study, Litha et al. [161] investigated PLA-PCL-graphene oxide (GO)-hydroxyapatite (HA) nanocomposites for biomedical implants. Their results showed that the synthesised GO and incorporation of both hydroxyapatite (HA) and graphene oxide (GO) showed promising implant properties. For example, good mechanical properties: the tensile strength was 42.9719 MPa, elongation at break was 2.2063%, and elastic modulus was 1416.98 MPa. These remarkable properties were assumed to be more suitable for biomedical implant applications. The work of Najera et al. [162] produced a 3D-printed PLA/PCL/TiO_2_ composite for bone replacement and grafting. The prepared composites were printed by fused deposition modelling (FDM). The authors used a 75/25 *w*/*w* PLA/PCL blend as their optimal composition and incorporated 1 wt.% of TiO_2_. Thermal analysis, mechanical analysis, and in vitro biocompatibility tests were performed to examine its credibility for bone replacement and grafting. There was an increase in the ultimate tensile strength of the PLA/PCL/TiO_2_ composites, suggesting that the nanoparticles interacted well with the polymer blend components. With the in vitro biocompatibility, the composites exhibited no cytotoxicity, meaning they showed no harmful effect on the cells. There are, however, very limited studies specifically on biodegradable PLA/PCL/TiO_2_/MCC micro-nanocomposites for the development of films for use as a barrier in personal hygiene applications. Our work focuses on filling this gap, with the purpose of determining their usefulness or suitability in real-life applications. These films could have the potential to replace petroleum-based polymers used in personal hygiene products. Figure 19 is a schematic diagram illustrating the optimal properties of polymer composites to produce ideal composite films for personal hygiene applications. Excellent interfacial adhesion between the filler and the polymer blend in PLA/PCL/TiO_2_/MCC composites would mean that the compatibility of the components in the blend would be improved. This would then lead to composites having good barrier properties (leakages would be prevented), and good mechanical properties, allowing the composites to be stretched or moved without tearing, which would guarantee comfort. Non-toxicity and antibacterial activity are essential properties in hygiene and health-related applications, to prevent infections and irritations upon usage. The importance of these composites has to do with how well these properties combine to provide a balance in the desired properties that are suitable for personal hygiene requirements. These requirements may include comfort and reliability.

## 8. Biocompatibility and Biodegradability of PLA/PCL Blends

**Figure 20:**
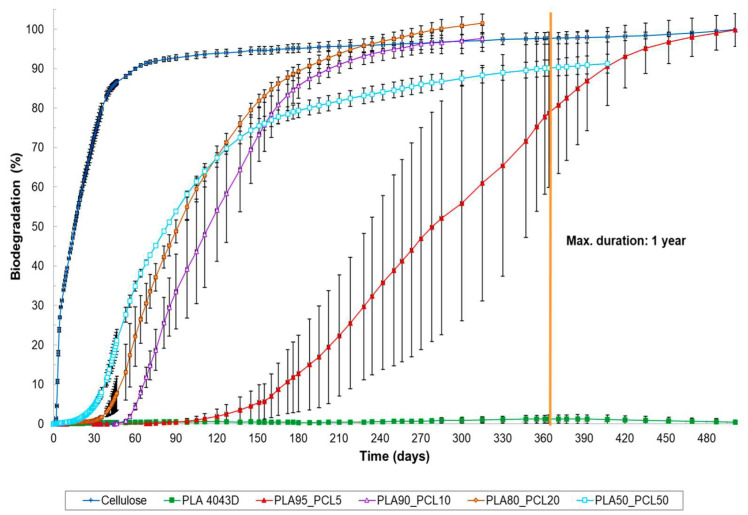
Biodegradation curves of cellulose (reference material), pure PLA, and PLA/PCL blends (95/5, 90/10, 80/20, 50/50 *w*/*w*) under home composting conditions (ISO 14855, 28 °C). The orange line indicates the maximum duration of all the materials in 1 year [164]. (Open access from ref. [164]. Copyright 2024, MDPI).

Biocompatibility and biodegradability are interconnected, and the properties of PLA/PCL blends with respect to them depend upon the blend ratio, morphology, molecular weights of polymers, processing, and environmental conditions, especially temperature [25,33,35,102,113]. These factors could allow the design of blends with custom-made degradation rates that are suitable for various applications. The difference between biocompatibility and biodegradability is that biocompatibility is the ability of materials to perform exceptionally well together, while biodegradability signifies the capacity of the material to undergo degradation in a controlled atmosphere to produce non-toxic byproducts. The results in previous studies have confirmed that PLA and PCL could behave as compatible polymers even though they are immiscible. Findings have also illustrated that having a higher PLA content in PLA/PCL blends could accelerate biodegradation while reducing flexibility. On the other hand, a higher content of PCL in PLA can improve flexibility while slowing down the degradation rate [25,102,113,163,164]. Solechan et al. [25], in their study, showed that the blend ratios of PLA/PCL blends could significantly affect the mechanical properties and biocompatibility. For example, having a higher PLA content in the blend (80/20 and 90/10 *w*/*w* PLA/PCL) led to immiscibility, seen by voids caused by detaching in an SEM analysis. The authors attributed this behaviour to enhanced mechanical strength at the 90/10 and 80/20 *w*/*w* compositions, which showed that the compatibility of these blends was improved. Narancic et al. [163] evaluated biodegradable PLA/PCL blends under home compositing conditions using ISO 14855 at 28 °C. In their study, the 80/20 *w*/*w* PLA/PCL blend ratio was used. The authors reported that the blend showed complete degradation within 60 days under home composting conditions. They also reported that the blend exceeded the biodegradation threshold of 90%. These results showed that blending PLA and PCL can be an effective approach for altering the biodegradability of polymers. The literature states that neat PLA generally degrades at temperatures above 50 °C under industrial composting conditions. Narancic et al.’s [163] research also shows that blending PLA with PCL allows biodegradation to occur at lower temperatures, and this serves as an energy saving mechanism. Similar observations were reported by Van de Perre et al. [164] when investigating the biodegradability of PLA/PCL blends under home compositing conditions (ISO 14855, 28 °C). In this study, 95/5, 90/10, 80/20, and 50/50 *w*/*w* PLA/PCL blends were evaluated. The authors reported that only the 80/20 *w*/*w* PLA/PCL blend exceeded the relative biodegradation threshold of 90%, as seen from Figure 20, and as also stipulated in the literature [163]. The results also indicated that the biodegradation percentage of the 80/20 *w*/*w* PLA/PCL blend exceeded 100%, which is higher than that of cellulose, which was used as the reference material for biodegradation.

## 9. Conclusions and Future Prospects

This work reviewed the morphology, thermal, thermomechanical, mechanical, barrier properties, and applications of PLA/PCL blends with the addition of sole fillers and hybrid fillers, with the intent of illustrating the gap in the literature with regard to their utilisation in personal hygiene products. In morphology studies, many researchers in their studies have observed that PLA/PCL blends are thermodynamically immiscible over a wide range of compositions. This behaviour was seen by polymers forming separate phases in the blend, due to differences in molecular weights and viscosities, the degree of crystallinity, and polarities, leading to phase separation, and to some extent, incompatibility. Despite the incompatibility between PLA and PCL, numerous studies have shown that the incorporation of a third component into the blend could improve compatibility between the two polymers. It has been well noted that filler localisation is another property that plays a big role in controlling the morphology of the polymer blends. In thermal and thermomechanical analyses, the results obtained by different authors in their respective studies corroborated the morphology results that PLA and PCL are thermodynamically immiscible. This was determined by the observation of two distinct thermal transitions (glass transition, melting, crystallisation, cold crystallisation temperatures and enthalpies) in DSC, and two separate transitions (storage and loss moduli, tan delta, glass transition temperature peaks) in DMA. In addition, the phase-separated morphology of the PLA/PCL blends brought about two distinct thermal degradation points and stabilities, which were visible by two degradation steps, as well as the stability towards the end of TGA analyses. The incorporation of nanofillers improved the interfacial adhesion between PLA and PCL and thus modified the thermal and thermomechanical properties of these blends. Moreover, combining fillers (hybrid fillers) or using a filler with a compatibiliser significantly improved the compatibility between PLA and PCL. Based on the numerous studies conducted, it was shown that combining constituents, namely, filler–filler and/or filler–compatibiliser in the PLA/PCL blends, led to significant improvement in the mechanical properties (elastic modulus, tensile strength, and elongation at break). The barrier properties of the PLA and PLA/PCL blends were also improved both in the presence of PCL and fillers. It has been proven in different studies that PLA and PCL are compatible even though they are immiscible. The biodegradation of the PLA/PCL blend at an 80/20 *w*/*w* ratio was found to exceed the relative biodegradation threshold of 90%. The blend’s biodegradation percentage was higher than that of cellulose, as the reference material under home composting conditions.

In the future, further research is required for the development of green biodegradable blend micro-nanocomposites that need to adhere to product standards, like having good biocompatibility, good thermal properties, being non-toxic and non-allergenic, and having good barrier and mechanical properties. Despite the potential advantages of PLA/PCL blend micro-nanocomposites, there is a shortage of reviews and experimental work on their processing, usage, and application in marketed products for personal hygiene. More work must be performed to develop materials that are sustainable in the long-term that will minimise the environmental impact caused by personal hygiene products.

## Figures and Tables

**Table 1:** Differences between biodegradable and petroleum-based polymers [14,15,16,17,18].

Biodegradable Polymers	Non-Biodegradable Polymers
Produced from renewable (corn and starch) and synthetic resources.	They are produced or synthesised from petroleum-based resources.
Environmentally friendly as they degrade naturally into water (H_2_O), carbon dioxide (CO_2_), and biomass.	They are not biodegradable and release toxic gases during incineration.
Consist of chains that can be hydrolytically or enzymatically cleaved.	Consist of non-polar covalent bonds, which are hard to break under the action of enzymes or water.
They last for a short period of time.	They are resistant to degradation and microbial attack, thus requiring a long duration for their decomposition.
Contain degradable groups in their polymeric backbone, like esters, amides, and ethers.	They consist of hydrocarbon chains.

**Table 2:** Different fillers, and their properties and versatile uses.

Type of Filler	Properties of Filler	Common Applications	References
Titanium dioxide (TiO_2_)	Antibacterial activity, low toxicity, photocatalytic activity, UV resistance, good mechanical and thermal properties.	Used in the removal of environmental pollutants, photocatalysis, cosmetics, medicine, and self-cleaning coatings.	[53,70,81]
Silicon dioxide (SiO_2_)	Fire resistant, high surface area, biocompatible, large band gap energy, non-toxic, and antimicrobial activity.	Rubber products, implants, coatings for ultrafiltration membranes (UF), chemical sensors, dental fillings, and cosmetics.	[74,82]
Cellulose and microcrystalline cellulose (MCC)	High specific surface area, biocompatibility, thermal stability, barrier properties, excellent mechanical strength, non-toxic nature, and antimicrobial activity.	Used to make membranes or filters for water purification, wound dressing, feminine hygiene products, dental implants, drug delivery, and packaging films.	[3,83]
Nanoclays	Excellent barrier, good mechanical and thermal stability.	Food package barrier films, rubber products, medicine, and cosmetics.	[84]
Zinc oxide nanoparticles (ZnO NPs)	Antimicrobial effect, UV shielding abilities, electromagnetic shielding.	Cosmetics, medicine (for wound healing), rubber products, and pigments.	[75,85]
Silver nanoparticles (AgNPs)	Strong antimicrobial agent, chemical stability, and good catalytic properties.	Used in wound dressings, cancer diagnosis, and surgical sutures.	[86]
Calcium carbonate (CaCO_3_)	High porosity, high surface area, non-toxic, and biocompatible.	Used in paints, pigments, paper coatings, and plastics.	[71,87]

**Table 3:** Selected studies on the morphologies of the PLA/PCL blends with varying blend compositions.

Sample Composition and Names	Processing Method	Influence of Varying Blend Composition on Morphology of the PLA/PCL Blend	Refs.
90/10, 80/20, 70/30 *w*/*w* PLA/PCL blends	Melt blending	PCL was evenly dispersed in the PLA phase in all the blends. It formed spherulite-shaped droplets, and they were observed in the continuous PLA phase.	[100]
98.75/1.25, 97.5/2.5, 95/5, 92.5/7.5 *w*/*w* PLA/PCL blends	Solution blending	PCL particles were dispersed in the PLA matrix. However, cracks and many ridges were seen in the blends, depicting plastic deformation prior to fracturing. Therefore, with the discussed behaviour, it is evident that there is poor compatibility between PLA and PCL.	[112]
90/10, 80/20, 70/30, 60/40, 50/50 *w*/*w* PLA/PCL blends	Melt mixing	It was reported that as PCL content increased, the particle size distribution also increased. At 30 wt.%, the particle size distribution of PCL enlarged. Furthermore, at a PCL content of 50 wt.%, the phase morphology exhibited a co-continuous structure.	[113]
80/20 *w*/*w* PLA/PCL blend	Batch mixer	Two-phase morphology was observed in the blend, indicating immiscibility between PLA and PCL.	[57]
90/10, 80/20, 70/30, 60/40 *w*/*w* PLA/PCL blends	Ball mill process	Immiscibility of the PLA/PCL blends was observed, which was visible by voids caused by the detachment of PCL particles from the PLA surface.	[25]
80/20 and 70/30 *w*/*w* PLA/PCL blends	Twin-screw extruder	The authors reported that both blends (80/20 and 70/30 *w*/*w*) exhibited a two-phase separation morphology irrespective of the incorporated PCL content. In addition, increasing PCL content resulted in an increased number of PCL droplets. Furthermore, at the 80/20 PLA/PCL blend, a sea-island morphology was observed.	[58]

**Table 4:** Tabulated surface energies of all the materials, as well as the calculated interfacial tensions of PLA/PCL, PLA/CN, and PCL/CN [60]. (Reproduced with permission from ref. [60]. Copyright 2020, John Wiley and Sons).

Surface Energies (mJm^−2^)				Interfacial Tension (mJm^−2^)	
	$\gamma_{s}$	$\gamma_{s}^{p}$	$\gamma_{s}^{d}$	$\gamma_{12}$	
PLA	52.3	10.2	42.0	PLA/PCL	2.20
PCL	51.1	16.1	34.9	PCL/CN	4.5
CN	68.9	28	40.9	PLA/CN	1.97

$\gamma_{s}$ represents total surface energy, $\gamma_{s}^{d}$ is the dispersive component of surface energy, $\gamma_{s}^{p}$ is a polar component of surface energy, and $\gamma_{12}\;\;$ is the interfacial tension between the components.

**Table 5:** Selected studies on the morphologies of PLA/PCL-filler micro-/nanocomposites with varying filler content.

Polymer Blend Composite	Weight Percentages (wt.%) of Filler	Technique Used to Analyse the Morphology	Remarks on the Influence of Fillers on the Morphology of the PLA/PCL-Filler Micro-/Nanocomposites.	Refs.
PLA/PCL/Zinc oxide nanoparticles (ZnO-NPs)	2.0, 4.0, and 6.0	FESEM and TEM	It was stated that the diameter of PCL droplets increased with increasing ZnO-NPs, from 610 nm in the blend to 775 nm in the nanocomposites with 6 wt.% ZnO-NPs. Furthermore, the wetting coefficient value was calculated as 2.09 mNm^−1^, which suggests that ZnO-NPs would preferentially localise within the PLA matrix.	[75]
PLA/PCL/Silicon carbide (SiC)	0.25, 0.5, 0.75, and 1.0		The introduction of SiC in the PLA/PCL blend showed good interfacial adhesion because SiC particles bonded well in a continuous PLA matrix. At a lower content of SiC (0.5 wt.%), there was a good dispersion of SiC in the PLA matrix. However, agglomeration of SiC was observed at a content above 0.5 wt.%.	[112]
PLA/PCL/Silk fibroin nanoparticles (SFNPs)	1.0		A continuous interface and uniform phase were observed with the incorporation of 1 wt.% content of SFNPs, which led to improved compatibility between PLA and PCL polymers. The improved compatibility resulted in a reduction in the PCL droplet size from 1.170 nm to 794 nm, which indicates a compatibilisation effect of SFNPs in the blend.	[61]
PLA/PCL/Silicon dioxide (SiO_2_)	1.0 and 3.0		Introducing 1 wt.% of SiO_2_ into the PLA/PCL blend (70/30) improved the compatibility between the two polymers. This behaviour was visible in two distinct phases in the blend without SiO_2_. Therefore, SiO_2_ acted as a compatibiliser by improving the interaction of PLA and PCL. Furthermore, agglomeration of SiO_2_ (3 wt.%) was visible in the PLA/PCL blend (50/50).	[120]
PCL/PLA/Montmorillonite (MMT)	1.0	TEM	It was noted that the presence of MMT significantly decreased the particle size of the PLA phase, and MMT was dispersed at the interface of the PCL/PCL blend, which could mean that the filler interacted with the two phases.	[73]

**Table 6:** Selected studies on thermal properties of polymer blends with/without the presence of fillers.

Polymer Blends	Filler Types	Remarks on Thermal Properties of the PLA/PCL Blend and/or Blend Composites	Refs.
PLA/PCL blends	Glycidyl methacrylate (GMA) and nanocalcium carbonate (NCC)	The presence of a compatibiliser (GMA) and nanoparticles (NCC) increased the degree of crystallinity of PLA in the PLA/PCL blends. This was seen by NCC acting as a nucleating agent, while GMA enhanced the crystallinity of the blends.	[65]
PLA/PCL blend (70/30 *w*/*w*)	Cellulose nanocrystals (CN)	The addition of CN did not affect PLA’s cold crystallisation (T_cc_). However, CN did improve the crystallisation (T_c_) of PCL in the blends. This behaviour could be related to CN being localised in the PCL phase and acting as a nucleating agent, facilitating the crystal growth of PCL. Furthermore, neither PCL nor CN influenced the melting temperature of the PLA polymer.	[60]
PLA/PCL blends	Nano-silica (SiO_2_)	Incorporating nano-silica slightly increased PLA’s melting temperature in the blends. The melting temperature of the PCL in the blends was reduced, but the exception was with 50% *w*/*w* PCL, where the melting temperature increased from 57 to 64 °C with 3 wt.% SiO_2_ content. Incorporating both PCL and nano-silica increased the degree of crystallinity of PLA. However, the only omissions are the blends of 60PLA/40PCL with 1, 2, and 3 wt.% SiO_2_ because both PCL and silica caused a reduction in the degree of crystallinity of PLA.	[120]
PLA/PCL blends	No filler	The blends showed that two individual endothermic peaks were detected, whereby the first peak was ascribed to the PCL endotherm and the second peak to the PLA endotherm. The observed results show that PLA and PCL are immiscible, which was confirmed by two endothermic peaks in the blend.	[129]
PLA/PCL blend	Multi-walled carbon nanotubes (CNTs) and montmorillonite (MMT)	It was stated that the presence of both CNTs and MMT slightly shifted the T_g_ of PCL towards higher temperatures, which could indicate improved compatibility between PLA and PCL in the blends. This behaviour was based on the unchanged T_g_ of PCL in the blend, which was attributed to poor compatibility between PCL and PLA.	[130]

**Table 7:** Mechanical properties of PLA composites [150]. (Reproduced with permission from ref. [150]. Copyright 2023, John Wiley and Sons).

Sample Name	Strength (MPa)	Elastic Modulus (GPa)	Elongation at Break (%)
PLA	49.4	1.5	3.79
PLA/PCL	37.17	1.25	11.72
PLA/PCL/5MCC	29.24	1.29	2.92
PLA/PCL/10MCC	22.25	1.31	2.31
PLA/PCL/15MCC	18.6	1.44	1.51
PLA/PCL/20MCC	16.42	1.52	1.15
PLA/PLAma/5MCC/ PCL	46.67	1.39	9.08

**Table 8:** Water vapour permeability values of the PLA-based blends [157]. (Reproduced with permission from ref. [157]; copyright 2024, Elsevier).

Sample	WVP (10^−14^ g·cm/cm^2^·s·Pa)
PLA	15.0 ± 0.2
PLA/poly(butylene-adipate-co-terephthalate (PBAT)	8.6 ± 0.1
PLA/polybutylene succinate (PBS)	10.1 ± 0.9
PLA/poly(3-hydroxybutyrate-co-4- Hydroxybutyrate (P34HB)	2.9 ± 0.6
PLA/polypropylene carbonate (PPC)	5.2 ± 0.4
PLA/PCL	3.1 ± 0.2

**Table 9:** Materials used in medical and hygiene products [5]. (Open access from ref. [5]. Copyright 2022, MDPI).

Medical and Hygiene Product	Materials Applied	Probable Biopolymer Replacement
Wound bandage	Polyvinyl alcohol (PVC), cotton.	PLA, PGA
Surgical masks	PET, cotton.	PLA, TPS
Disposable diapers	Polyacrylic acid, PVA copolymers.	TPS, PLA

## Data Availability

Not applicable.
